# Adolescent mental well-being, religion and family activities: a cross-sectional study (Northern Ireland Schools and Wellbeing Study)

**DOI:** 10.1136/bmjopen-2023-071999

**Published:** 2023-06-22

**Authors:** Jordan Bamford, Gerard Leavey, Michael Rosato, Natalie Divin, Gavin Breslin, Dagmar Corry

**Affiliations:** 1 Division of Psychology and Mental Health, The University of Manchester, Manchester, UK; 2 Bamford Centre for Mental Health and Wellbeing, University of Ulster, Coleraine, UK; 3 School of Psychology, Ulster University, Coleraine, UK; 4 School of Psychology, Queen's University Belfast, Belfast, UK

**Keywords:** MENTAL HEALTH, Child & adolescent psychiatry, PSYCHIATRY

## Abstract

**Objectives:**

In this study, we seek to explore the relationship between adolescent mental well-being, religion and family activities among a school-based adolescent sample from Northern Ireland.

**Setting:**

The Northern Ireland Schools and Wellbeing Study is a cross-sectional study (2014–2016) of pupils in Northern Ireland aged 13–18 years.

**Participants:**

1618 adolescents from eight schools participated in this study.

**Outcomes measures:**

Our primary outcome measure was derived using the Warwick-Edinburgh Mental Wellbeing Scale. We used hierarchical linear regression to explore the independent effects of a range of personal/social factors, including religious affiliation, importance of religion and family activities.

**Results:**

In fully adjusted models, older adolescents and females reported lower mental well-being scores—for the year-on-year increase in age β=−0.45 (95% CI=−0.84, –0.06), and for females (compared with males) β=−5.25 (95% CI=−6.16, –4.33). More affluent adolescents reported better mental well-being. No significant differences in mental well-being scores across religious groups was found: compared with Catholics, Protestant adolescents recorded β=−0.83 (95% CI=−2.17, 0.51), other religious groups β=−2.44 (95% CI=−5.49, 0.62) and atheist adolescents β=−1.01 (95% CI=−2.60, 0.58). The importance of religion in the adolescents’ lives was also tested: (compared with those for whom it was not important) those for whom it was very important had better mental well-being (β=1.63: 95% CI=0.32, 2.95). Higher levels of family activities were associated with higher mental well-being: each unit increase in family activity produced a 1.45% increase in the mental well-being score (β=0.78: 95% CI=0.67, 0.90).

**Conclusions:**

This study indicates that non-religious adolescents may have lower mental well-being scores when compared with their more religious peers, irrespective of religious denomination. This may relate to both a sense of lack of firm identity and perceived marginalisation. Additionally, adolescents with poor family cohesion are more vulnerable to poor mental well-being.

**STRENGTHS AND LIMITATIONS OF THIS STUDY**

- This study uses well-validated instruments to determine personal and social factors associated with mental well-being.
- This study uses a representative sample of adolescents from a range of schools.
- A limitation is that the study is cross-sectional, and therefore causality cannot be demonstrated.

## Introduction

### Adolescent mental health

Worldwide, mental disorders—particularly mood disorders—among children and adolescents worldwide may be increasing, something noted even before the recent COVID-19 pandemic.^1–5^ In England, one National Health Service (NHS) survey found that 14.4% of 11–16 year olds reported a mental disorder,^5^ and evidence suggests increased demand over the last decade for specialist mental health interventions for children and adolescents.^6^ Additionally, there is evidence of increasing self-harm among young people, one study shows a 68% increase among girls aged 13–16 years since 2011.^7^ In the UK, reported suicide has increased in those less than 18 years of age, mainly driven by an increase in females aged 16 years and males aged 17 years.^8^

Adolescence is a critical life-course period for health, with consequent mental health problems having a much greater long-term cost than those developed during adulthood.^9–11^ The brain is particularly sensitive to stressors during this time.^12^ Social isolation, self-esteem and connectedness are important determinants of adolescent mental health.^13^ Associations between depressive symptoms and social media use have been identified, and this apparent vulnerability is greater for girls than boys.^14^ Furthermore, among adolescents, sex, social deprivation, child-in-need status, ethnicity and age all predict poor mental health outcomes.^15 16^

### Mental well-being, religion and family activities

Mental well-being is the positive aspect of mental health, relating to psychological functioning, life-satisfaction and ability to develop and maintain mutually beneficial relationships.^17^ The dual-continua model of mental health suggests that mental illness and mental well-being are not extreme ends of a single spectrum but rather they reflect distinct continua which influence one another via complex interrelationships.^18^ Among adults, better mental well-being is associated with reduced risks of developing mental illness and improved chance of recovery from mental illness.^19 20^ Evidence suggests that mental well-being is positively associated with a broad range of positive mental and physical health outcomes among adolescents in the UK.^21^

Evidence suggests that greater religiosity (mainly religious service attendance) is associated with higher well-being among adults,^22 23^ although this evidence is derived mainly from predominantly Christian groups. One natural experiment suggests that greater involvement in religious activity (Ramadan fasting) increases well-being among Muslims.^24^ Evidence suggests religiosity and spirituality are beneficial for adolescents: correlating with positive health attitudes and behaviours^25^ and having a positive effect on psychological outcomes including risk behaviour, depression, well-being and self-esteem.^26^ It is thought that attendance at religious services can bolster mental well-being by reducing loneliness, providing social support and engagement with community^27 28^; and evidence from the UK also indicates that more frequent attendance improves well-being among adults.^29^ However, religion can be damaging to mental well-being,^30^ particularly for vulnerable groups such as lesbian, gay or bisexual adolescents.^31^ Another key determinant of mental well-being is within-family group activities, which evidence suggests is both an independent predictor of adolescent well-being,^32^ and a measure of family cohesion. The family has both a role in moderating risk factors for poor child development^33^ and influences the building of resilience.^34^

### The context in Northern Ireland

Adolescent mental health in Northern Ireland (NI) is a public health concern following increased suicidality among young people.^21 35–37^ Previous research suggests that post-conflict generations who have experienced violence in their communities may be at higher risk of depression, psychotic symptoms or substance misuse.^38^ Religious affiliation is of continuing social importance since the putative ending of the civil conflict (The Troubles). The most recent census data (from 2021) shows that NI comprises a small majority Catholic (45.7%) and large minority Protestant group (43.5%), with 1.5% identifying as other non-Christian, and the remaining 9.3% of the population identifying as not belonging to any religion—with this latter group increasing from 5.6% in 2011.^39^ There is evidence that minority religious and atheist groups report poorer mental health and increased suicidality.^36^ Loneliness and poverty may also be associated with poor mental health in this population.^40^

In this study, we explore the relationship between adolescent mental well-being, religion and family cohesion. Such findings offer useful insights to inform child and adolescent mental health services and guide policy.

## Methods

### Study design and setting

This is a cross-sectional study (2014–2016) of pupils in NI aged 13–18 years, based on a questionnaire and linked school administrative records data.^36 41^ Eight schools were selected from a database of post-primary schools based on locale (urban/rural) and school type (grammar or secondary modern). While grammar school enrolment is based on selection (through examination), it is generally acknowledged to have a strong middle-class bias. In NI, 43% of post-primary children attend grammar schools.^42 43^ As part of the study protocol, parents received a letter explaining the study, with an opt-out form for those requesting non-participation. All pupils provided written informed consent. Only five parents or children declined to participate. School absence was between 5% and 8% across the selected schools. Pupils completed the questionnaires in class groups following procedures described previously.^44^ A copy of the questionnaire is included as online supplemental file 1. Our sample comprised of 1618 young people who provided complete responses to the questionnaire.

10.1136/bmjopen-2023-071999.supp1 Supplementary data

### Measures

#### Outcome variable

Mental well-being: the Warwick-Edinburgh Mental Wellbeing Scale is a widely used measures of mental well-being,^45 46^ in both NHS and private health services. Overall, it is deemed a valid and reliable tool for measuring well-being in diverse populations.^47 48^ It comprises 14 items covering psychological functioning and subjective well-being, each a positively worded question on a five-point Likert scale. The responses are summed to provide a total score which ranges between 14 and 70, with higher scores indicating better mental well-being.

#### Measures of religion

Religious group: participants indicated which religious group they identified with—summarised as Catholic, Protestant, other faith group or atheist/agnostic.

Importance of religion: participants were asked about ‘how important is religion to your family’—with responses very important, moderately important or not important.

#### Family activities

To assess family cohesion, we used the Family Activities scale^49^—six questions, each a five-point Likert scale (with responses 1=never to 5=every day). The responses are summed, with high scores indicating an integrated family life.

#### Confounders

We controlled for age (continuous) and ethnicity (white European, other ethnic group). Sex (male, female)—rather than gender—was recorded, as requested by school principals. We controlled for school type (grammar, non-grammar) and family affluence—the latter measured using the family affluence scale which quantifies socioeconomic inequity. This measure is reliable, sensitive and validated for differentiating levels of affluence.^50^ It was used in analysis as a continuous variable (range=0 to 9), with higher scores indicating greater affluence. We also controlled for the locale of the school (rural, urban).

### Analysis

Characteristics of the population were determined, and the relationship with characteristics of the population and mental well-being examined, with mean and SD presented. To assess for significant differences between means, we used t-tests and analysis of variances (for binary or categorical variables, respectively). For continuous variables, means and SD are presented. We used hierarchical linear regression to explore the influence of religion, religious importance and family activities on well-being—and present results as beta coefficient, associated 95% CIs and p values.

### Patient and public involvement

None.

## Results

Population characteristics are presented in table 1. The sample included adolescents aged 13 to 18 years, with over 90% aged 16 years or younger. This sample was mainly female (62.7%); predominantly white European (94.7%), with 57.5% attending non-grammar schools. With family affluence, the mean score was 6.0 (SD 1.7); while for family activities the mean was 16.6 (SD 3.8). Most adolescents identified as Catholic (60.4%) and 10.3% identified as being atheist/agnostic; and over 70% said that religion was either moderately or very important to their family. In this sample, 60.3% of adolescents attended school in an urban area.

**Table 1:** Sample characteristics and unadjusted distribution of well-being

	Whole sample	WEMWBS score	Significance
	% (n)	Mean (SD)	
Age			
13	11.5 (186)	50.1 (8.5)	0.203
14	30.4 (492)	48.4 (9.5)	
15	25.7 (416)	47.1 (10.3)	
16	24.4 (395)	46.9 (9.8)	
17	7.2 (116)	46.0 (9.4)	
18	0.8 (13)	47.6 (8.6)	
Sex			
Male	37.3 (603)	51.0 (8.4)	<0.001
Female	62.7 (1015)	45.7 (9.9)	
Ethnicity			
White European	94.7 (1532)	47.7 (9.8)	0.188
Other	5.3 (86)	47.9 (8.7)	
Religious grouping			
Catholic	60.4 (978)	47.8 (9.6)	<0.001
Protestant	27.1 (439)	48.4 (9.4)	
Other	2.1 (34)	46.1 (11.3)	
Atheist/agnostic	10.3 (167)	45.4 (10.8)	
Importance of religion			
Very	19.8 (321)	49.3 (10.1)	0.03
Moderately	51.2 (829)	48.0 (9.1)	
Unimportant	28.9 (468)	46.1 (10.3)	
School type			
Grammar	42.5 (688)	46.7 (10.1)	0.004
Non-grammar	57.5 (930)	48.4 (9.4)	
Rurality			
Rural	39.7 (643)	47.0 (10.0)	<0.001
Urban	60.3 (975)	48.8 (9.3)	

Continuous variables			
	Mean (SD)	Beta coefficient	P value
Family activities			
Range 6–30	16.6 (3.8)	0.88	<0.001
Family affluence			
Range 0–9	6.0 (1.7)	0.63	<0.001
Mental well-being			
Range 14–70	47.7 (9.7)	–	–

WEMWBS, Warwick-Edinburgh Mental Wellbeing Scale.

The mean score for mental well-being over the whole sample was 47.7 (SD 9.7). We identified no significant difference across age group and ethnic group with regard to mental well-being (unadjusted for other confounding predictors). We identified that across the sexes, religious groups, religious importance to family, rurality and school type, there were significant differences in mean scores for mental well-being (unadjusted for other confounding variables). We identified that females had on average lower mental well-being scores compared with males, 45.7 (SD 9.9) and 51.0 (SD 8.4), respectively. Lower well-being scores were found among atheist/agnostic adolescents and those adolescents whose families did not consider religion to be important. Adolescents in grammar schools had lower on average mental well-being scores, as too did those adolescents attending rural schools. We identified that higher scores for both the Family Act Scale and the Family Influence Scale were associated with greater well-being scores for adolescents.

Table 2 presents results for four incrementally adjusted models (M1 to M4) examining the association between well-being and the selected risk factors. In M1, both older and female adolescents record significantly poorer mental well-being. More affluent adolescents recorded better mental well-being. Neither grammar school status, rurality or ethnicity were significantly associated with predicting mental well-being.

**Table 2:** Relationship between mental well-being and included demographic and family characteristics

	M1 Beta coefficient (95% CI)	M2 Beta coefficient (95% CI)	M3 Beta coefficient (95% CI)	M4 Beta coefficient (95% CI)
Age†	−0.82 (−1.23, –0.42)**	−0.75 (−1.16, –0.34)**	−0.76 (−0.17, –0.35)**	−0.45 (−0.84, –0.06)*
Sex				
Female (ref=male)	−5.21 (−6.17, –4.25)**	−5.41 (−6.37, –4.44)**	−5.38 (−6.34, –4.41)**	−5.25 (−6.16, –4.33)**
Ethnicity				
(ref=white European)				
Other	−0.64 (−2.66, 1.39)	−0.23 (−2.27, 1.81)	−0.32 (−2.35, 1.72)	−0.29 (−2.22, 1.65)
School type				
(ref=grammar)				
Non-grammar	0.00 (−1.37, 1.38)	0.79 (−1.00, 2.58)	0.94 (−0.85, 2.73)	1.23 (−0.46, 2.93)
Family affluence†	0.76 (0.48, 1.03)**	0.75 (0.48, 1.02)**	0.70 (0.43, 0.97)**	0.41 (0.14, 0.67)*
Locale of residence				
Rural (ref=urban)	0.99 (−0.31, 2.29)	0.31 (−1.19, 1.80)	0.13 (−1.37, 1.63)	−0.61 (−2.04, 0.82)
Religion				
(ref=Catholic)				
Protestant		–0.82 (–2.23, 0.59)	–0.60 (–2.01, 0.81)	–0.83 (–2.17, 0.51)
Other		–2.95 (–6.18, 0.28)	–3.05 (–6.28, 0.16)	–2.44 (–5.49, 0.62)
Atheist		–2.98 (–4.57, –1.40)**	–2.06 (–3.73, 0.40)*	–1.01 (–2.60, 0.58)
Importance of religion				
(ref=not important)				
Moderately			1.49 (0.37, 2.61)*	0.91 (–0.16, 1.9)
Very			2.56 (1.19, 3.94)**	1.63 (0.32, 2.95)*
Family activities†				0.78 (0.67, 0.90)**

Data represents beta coefficients (and 95% CIs) from four incrementally developed regression models.

*<0.05; **<0.001.

†Variable treated as continuous.

figures in bold repressents statistical significance

The addition of religious group (M2) did not materially influence the already established associations of sex, age and affluence with mental well-being. However, atheist adolescents (compared with Catholics) reported significantly poorer mental well-being (β=−2.98: 95% CI=−4.57, –1.40). No significant differences between the reference Catholics and Protestant or adolescents from other religious groups ere identified.

With the inclusion of importance of religion (M3), the established associations between well-being and age, sex and family affluence persisted, and while the poorer well-being associated with non-religious adolescents was again evident, its impact on poorer well-being was reduced—from β=−2.98 (95% CI=−4.57, –1.40) to β=−2.06 (95% CI=−3.73, –0.40) in M2 and M3, respectively. Adolescents whose family considered religion to be either moderately or very important were (compared with those for whom religion was of no importance), reported significantly better well-being, for example, β=2.56 (95% CI=1.19, 3.94), seen in those for whom religion was very important.

Finally, M4—adding family activity levels—shows the fully adjusted model. Again, the influence of sex and age persists, as too does family affluence (though with reduced beta coefficients). Inclusion of family activity levels also: (a) obviates any association between all religious denomination and well-being—including the non-religious group; and (b) constrains any beneficial effects of the importance of religion, and only those adolescents where religion was very important retain any beneficial effect (β=1.63: 95% CI=0.32, 2.95). Family activity levels are associated with significant higher mental well-being—β=0.78 (95% CI=0.67, 0.90), a per-unit increase of 1.45% well-being.

## Discussion

In this study, examining predictors of mental well-being in a school-based sample of adolescents, we found that in families where religion was not important, there were poorer adolescent well-being outcomes scores compared with their peers for whom religion was very important. This relationship has been identified in other studies which explored the influence of religious importance and its impact independent of religious affiliation.^51^ We initially identified that atheist/agnostic adolescents had lower mental well-being scores compared with other adolescent groups (catholic/protestant/other groups); however, when we accounted for differences in family activities, difference of mental well-being scores across religious groups were insignificant. Thus, where previous research has found non-religious groups having better mental health outcomes, this is not evident among our sample.^52^ Previous research using this sample found evidence of increased suicidality and mental illness associated with non-religious adolescents^36^—this apparent vulnerability may relate to social and psychological challenges associated with both being different and a felt lack of firm identity. This experience of marginalisation associated with non-religious groupings has been found to contribute to poorer mental health outcomes in the USA.^53 54^

In this sample, females (compared with males) experienced poorer well-being: female vulnerability has been identified elsewhere: one study of Mexican adolescents found that post-menarche females recorded increased depressive symptoms, externalising problems and negative body image^55^; and another study identified that older female adolescents reported lower levels of self-compassion and lower emotional well-being.^56^ Male/female differences in well-being have been related to societal cultural attitudes and inequity surrounding gender.^57^

In this study, more cohesive family activity levels were key to better mental well-being. Adolescents with secure attachments and cohesive families have been found to engage less in high-risk behaviours, have fewer mental health problems, enhanced social skills and better coping strategies.^58^ Some evidence suggests that parenting style and, by implication, family activity levels play a key role in emerging adult mental health, particularly for females.^59^

In the fully adjusted model, high levels of affluence were significantly associated with better well-being. In an earlier study using this, data affluence had little influence on either mental health outcomes or trust in GPs.^36^ Family affluence may therefore be associated with mental well-being, but not necessarily mental health outcomes. Other research has shown that mental well-being and mental illness have distinct and different correlates and that actual and perceived socioeconomic circumstance may correlate differently from each other.^60^ Other research has shown that socioeconomic position was associated with self-harm among adolescents, mainly for girls,^61^ and a systematic review found lower socioeconomic status associated with mental health problems.^62^

### Strengths and limitations

This study uses well-validated instruments to determine personal and social factors associated with mental well-being. This sample of adolescents from eight schools is relatively representative, with similar proportions of adolescents in grammar school education,^43^ and a similar proportion of atheist/agnostic adolescents compared with the general NI population.^39^ While we note that Catholics are over-represented in this sample (60% from a Catholic background, compared with 45.7% in the general population)^39^—current demographic trends in post-primary school enrolment record higher proportions of adolescent Catholics: in 2021/2022, 50.2% of the grammar school population (32 990/65 313) and 51.7% (44 795/86 588) of the non-grammar school population were Catholic.^43^ This age-related redistribution of Catholics in relation to Protestants in NI mirrors current local demographic trends.^63^ Furthermore, our sample contains 5.3% of adolescents who do not belong to white European ethnicity. Census data shows that 3.4% of the population are from minority ethnic groups in NI and tend to be younger which is in keeping with our sample.^64^

A limitation is that the study is cross-sectional, and therefore causality cannot be inferred. Further we recognise that the use of self-reported data has limitations, with social desirability bias possibly impacting on the results. Finally, as previously mentioned, we measured sex (not gender) at the request of school principals—we therefore acknowledge that this study cannot explore the influence of gender identity problems on mental well-being.

## Conclusion

This study suggests that religious practice and family activities are important determinants of adolescent well-being. We highlight vulnerabilities associated with adolescents for whom religion is not important, in relation to the experience of marginalisation and perceived lack of a firm identity. Additionally, the study suggests that less cohesive family units may also present vulnerabilities which can impact poor adolescent mental well-being. Future policy should be mindful of these personal and social factors, and child and adolescent mental health services in NI should consider the increased vulnerability of non-religious youth.

## Supplementary Material

Reviewer comments

Author's manuscript

## Data Availability

An anonymised, de-identified database can be made available on request. Not applicable.

## References

[1] Racine N, McArthur BA, Cooke JE, et al. Global prevalence of depressive and anxiety symptoms in children and adolescents during COVID-19: a meta-analysis. JAMA Pediatr 2021;175:1142–50. 10.1001/jamapediatrics.2021.2482 34369987 PMC8353576

[2] Dalsgaard S, Thorsteinsson E, Trabjerg BB, et al. Incidence rates and cumulative incidences of the full spectrum of diagnosed mental disorders in childhood and adolescence. JAMA Psychiatry 2020;77:155. 10.1001/jamapsychiatry.2019.3523 31746968 PMC6902162

[3] Lu W. Adolescent depression: national trends, risk factors, and healthcare disparities. Am J Health Behav 2019;43:181–94. 10.5993/AJHB.43.1.15 30522576

[4] Polanczyk GV, Salum GA, Sugaya LS, et al. Annual research review: a meta‐analysis of the worldwide prevalence of mental disorders in children and adolescents. J Child Psychol Psychiatry 2015;56:345–65. 10.1111/jcpp.12381 25649325

[5] Sadler K, Vizard T, Ford T, et al. Mental health of children and young people in England, 2017. 2018.

[6] Sarginson J, Webb RT, Stocks SJ, et al. Temporal trends in antidepressant prescribing to children in UK primary care, 2000-2015. J Affect Disord 2017;210:312–8. 10.1016/j.jad.2016.12.047 28068620 PMC5458802

[7] Morgan C, Webb RT, Carr MJ, et al. Incidence, clinical management, and mortality risk following self harm among children and adolescents: cohort study in primary care. BMJ 2017;359:j4351. 10.1136/bmj.j4351 29046278 PMC5641980

[8] Manchester Uo. The National confidential inquiry into suicide and safety in mental health. Annual report: UK patient and general population data, 2009-2019, and real time surveillance data. Available: https://sites.manchester.ac.uk/ncish/reports/annual-report-2022 [Accessed 17 Jan 2023].

[9] Lee FS, Heimer H, Giedd JN, et al. Adolescent mental health—opportunity and obligation. Science 2014;346:547–9. 10.1126/science.1260497 25359951 PMC5069680

[10] Moore GF, Cox R, Evans RE, et al. School, peer and family relationships and adolescent substance use, subjective wellbeing and mental health symptoms in Wales: a cross sectional study. Child Indic Res 2018;11:1951–65. 10.1007/s12187-017-9524-1 30524519 PMC6244918

[11] Patton GC, Sawyer SM, Santelli JS, et al. Our future: a Lancet commission on adolescent health and wellbeing. Lancet 2016;387:2423–78. 10.1016/S0140-6736(16)00579-1 27174304 PMC5832967

[12] Romeo RD. The impact of stress on the structure of the adolescent brain: implications for adolescent mental health. Brain Res 2017;1654:185–91. 10.1016/j.brainres.2016.03.021 27021951

[13] Preston AJ, Rew L. Connectedness, self-esteem, and prosocial behaviors protect adolescent mental health following social isolation: a systematic review. Issues Ment Health Nurs 2022;43:32–41. 10.1080/01612840.2021.1948642 34346800

[14] Kelly Y, Zilanawala A, Booker C, et al. Social media use and adolescent mental health: findings from the UK millennium cohort study. EClinicalMedicine 2018;6:59–68. 10.1016/j.eclinm.2018.12.005 31193561 PMC6537508

[15] Deighton J, Lereya ST, Casey P, et al. Prevalence of mental health problems in schools: poverty and other risk factors among 28 000 adolescents in England. Br J Psychiatry 2019;215:565–7. 10.1192/bjp.2019.19 30698513 PMC7557860

[16] Bøe T, Skogen JC, Sivertsen B, et al. Economic volatility in childhood and subsequent adolescent mental health problems: a longitudinal population-based study of adolescents. BMJ Open 2017;7:e017030. 10.1136/bmjopen-2017-017030 PMC5623474 28928191

[17] Stewart-Brown S, Janmohamed K. Warwick-Edinburgh mental well-being scale. User guide Version 1; 2008.

[18] Iasiello M, van Agteren J, Cochrane EM. Mental health and/or mental illness: a scoping review of the evidence and implications of the dual-Continua model of mental health. Evidence Base 2020;2020:1–45. 10.21307/eb-2020-001

[19] Keyes CLM, Dhingra SS, Simoes EJ. Change in level of positive mental health as a predictor of future risk of mental illness. Am J Public Health 2010;100:2366–71. 10.2105/AJPH.2010.192245 20966364 PMC2978199

[20] Iasiello M, van Agteren J, Keyes CLM, et al. Positive mental health as a predictor of recovery from mental illness. J Affect Disord 2019;251:227–30. 10.1016/j.jad.2019.03.065 30927584 PMC6487880

[21] McKay MT, Andretta JR, Cole JC, et al. Socio-demographic predictors of well-being in United Kingdom adolescents, and the impact of well-being on a range of health-related outcomes. Psychiatry Research 2020;285:112728. 10.1016/j.psychres.2019.112728 31870619

[22] VanderWeele TJ. Religion and health in Europe: cultures, countries, context. Eur J Epidemiol 2017;32:857–61. 10.1007/s10654-017-0310-7 28884408 PMC5681407

[23] Chen Y, VanderWeele TJ. Associations of religious upbringing with subsequent health and well-being from adolescence to young adulthood: an outcome-wide analysis. Am J Epidemiol 2018;187:2355–64. 10.1093/aje/kwy142 30215663 PMC6211237

[24] Campante F, Yanagizawa-Drott D. Does religion affect economic growth and happiness? Evidence from Ramadan. Q J Econ 2015;130:615–58. 10.1093/qje/qjv002

[25] Rew L, Wong YJ. A systematic review of associations among religiosity/spirituality and adolescent health attitudes and behaviors. J Adolesc Health 2006;38:433–42. 10.1016/j.jadohealth.2005.02.004 16549305

[26] Yonker JE, Schnabelrauch CA, Dehaan LG. The relationship between spirituality and religiosity on psychological outcomes in adolescents and emerging adults: a meta-analytic review. J Adolesc 2012;35:299–314. 10.1016/j.adolescence.2011.08.010 21920596

[27] Lim C, Putnam RD. Religion, social networks, and life satisfaction. Am Sociol Rev 2010;75:914–33. 10.1177/0003122410386686

[28] Bhui K, King M, Dein S, et al. Ethnicity and religious coping with mental distress. J Ment Health 2008;17:141–51. 10.1080/09638230701498408

[29] Aksoy O, Bann D, Fluharty ME, et al. Religiosity and mental wellbeing among members of majority and minority religions: findings from understanding society: the UK household longitudinal study. Am J Epidemiol 2022;191:20–30. 10.1093/aje/kwab133 33977294 PMC8751808

[30] Weber SR, Pargament KI. The role of religion and spirituality in mental health. Curr Opin Psychiatry 2014;27:358–63. 10.1097/YCO.0000000000000080 25046080

[31] Page MJL, Lindahl KM, Malik NM. The role of religion and stress in sexual identity and mental health among Lesbian, gay, and Bisexual youth. J Res Adolesc 2013;23:665–77. 10.1111/jora.12025 PMC3828207 24244081

[32] Maynard MJ, Harding S. Ethnic differences in psychological well-being in adolescence in the context of time spent in family activities. Soc Psychiatry Psychiatr Epidemiol 2010;45:115–23. 10.1007/s00127-009-0047-z 19350190 PMC2803750

[33] Clarke-Stewart A, Dunn J. Families count: Effects on child and adolescent development. Cambridge University Press, 2006. 10.1017/CBO9780511616259

[34] Luthar SS, Cicchetti D, Becker B. The construct of resilience: a critical evaluation and guidelines for future work. Child Dev 2000;71:543–62. 10.1111/1467-8624.00164 10953923 PMC1885202

[35] McAlister S, Haydon D, Scraton P. Violence in the lives of children and youth in "post-conflict" Northern Ireland. Child Youth Serv 2013;23:1–22. 10.1353/cye.2013.0001

[36] Leavey G, Rosato M, Harding S, et al. Adolescent mental health problems, suicidality and seeking help from general practice: a cross-sectional study (Northern Ireland schools and wellbeing study). J Affect Disord 2020;274:535–44. 10.1016/j.jad.2020.05.083 32663986

[37] Carlisle PA. We don't talk about that around here": religion, spirituality and mental health in Northern Ireland. Ment Health Relig Cult 2015;18:396–407. 10.1080/13674676.2015.1071787

[38] McAloney K, McCrystal P, Percy A, et al. Damaged youth: prevalence of community violence exposure and implications for adolescent well‐being in post‐conflict Northern Ireland. J Community Psychol 2009;37:635–48. 10.1002/jcop.20322

[39] NISRA. Main statistics for Northern Ireland statistical bulletin: religion. 2022. Available: https://wwwnisragovuk/system/files/statistics/census-2021-main-statistics-for-northern-ireland-phase-1-statistical-bulletin-religionpdf

[40] Shevlin M, Murphy S, Mallett J, et al. Adolescent loneliness and psychiatric morbidity in Northern Ireland. Br J Clin Psychol 2013;52:230–4. 10.1111/bjc.12018 24215150

[41] Corry DAS, Leavey G. Adolescent trust and primary care: help-seeking for emotional and psychological difficulties. J Adolesc 2017;54:1–8. 10.1016/j.adolescence.2016.11.003 27838545

[42] Cribb J, Jesson D, Sibieta L, et al. Poor grammar: entry into grammar schools for disadvantaged pupils in England. Sutton Trust, 2013.

[43] NISRA. Annual Enrolments at schools and in funded pre-school education in Northern Ireland. 2022. Available: https://wwweducation-nigovuk/sites/default/files/publications/education/Revised%2011th%20March%202022%20-%20Annual%20enrolments%20at%20schools%20and%20in%20funded%20pre-school%20education%20in%20Northern%20Ireland%2C%202021-22pdf

[44] Leavey G, Rothi D, Paul R. Trust, autonomy and relationships: the help-seeking preferences of young people in secondary level schools in London (UK). J Adolesc 2011;34:685–93. 10.1016/j.adolescence.2010.09.004 20952053

[45] Ng Fat L, Scholes S, Boniface S, et al. Evaluating and establishing national norms for mental wellbeing using the short Warwick–Edinburgh mental well-being scale (SWEMWBS): findings from the health survey for England. Qual Life Res 2017;26:1129–44. 10.1007/s11136-016-1454-8 27853963 PMC5376387

[46] Anthony R, Moore G, Page N, et al. Measurement Invariance of the short Warwick-Edinburgh mental wellbeing scale and latent mean differences (SWEMWBS) in young people by current care status. Qual Life Res 2022;31:205–13. 10.1007/s11136-021-02896-0 34050443 PMC8800901

[47] Stewart-Brown S, Platt S, Tennant A, et al. The Warwick-Edinburgh mental well-being scale (WEMWBS): a valid and reliable tool for measuring mental well-being in diverse populations and projects. J Epidemiology Community Health 2011;65:A38–9. 10.1136/jech.2011.143586.86

[48] Tennant R, Hiller L, Fishwick R, et al. The Warwick-Edinburgh mental well-being scale (WEMWBS): development and UK validation. Health Qual Life Outcomes 2007;5:63. 10.1186/1477-7525-5-63 18042300 PMC2222612

[49] Harding S, Read UM, Molaodi OR, et al. The determinants of young adult social well-being and health (DASH) study: diversity, Psychosocial determinants and health. Soc Psychiatry Psychiatr Epidemiol 2015;50:1173–88. 10.1007/s00127-015-1047-9 25861790 PMC4519637

[50] Currie CE, Elton RA, Todd J, et al. Indicators of socioeconomic status for adolescents: the WHO health behaviour in school-aged children survey. Health Educ Res 1997;12:385–97. 10.1093/her/12.3.385 10174221

[51] Green M, Elliott M. Religion, health, and psychological well-being. J Relig Health 2010;49:149–63. 10.1007/s10943-009-9242-1 19283486

[52] Baker JO, Stroope S, Walker MH. Secularity, religiosity, and health: physical and mental health differences between atheists, agnostics, and nonaffiliated theists compared to religiously affiliated individuals. Soc Sci Res 2018;75:44–57. 10.1016/j.ssresearch.2018.07.003 30080491

[53] Brewster ME, Velez BL, Geiger EF, et al. It’s like herding cats: atheist minority stress, group involvement, and psychological outcomes. J Couns Psychol 2020;67:1–13. 10.1037/cou0000392 31697119

[54] Doane MJ, Elliott M. Perceptions of discrimination among atheists: consequences for Atheist identification, psychological and physical well-being. Psycholog Relig Spiritual 2015;7:130–41. 10.1037/rel0000015

[55] Benjet C, Hernandez-Guzman L. Gender differences in psychological well-being of Mexican early adolescents. Adolescence 2001;36:47–65. 11407635

[56] Bluth K, Campo RA, Futch WS, et al. Age and gender differences in the associations of self-compassion and emotional well-being in a large adolescent sample. J Youth Adolesc 2017;46:840–53. 10.1007/s10964-016-0567-2 27632177 PMC5346326

[57] Tesch-Römer C, Motel-Klingebiel A, Tomasik MJ. Gender differences in subjective well-being: comparing societies with respect to gender equality. Soc Indic Res 2007;85:329–49. 10.1007/s11205-007-9133-3

[58] Moretti MM, Peled M. Adolescent-parent attachment: bonds that support healthy development. Paediatr Child Health 2004;9:551–5. 10.1093/pch/9.8.551 19680483 PMC2724162

[59] Steele EH, McKinney C. Emerging adult psychological problems and parenting style: moderation by parent-child relationship quality. Pers Individ Differ 2019;146:201–8. 10.1016/j.paid.2018.04.048

[60] Patalay P, Fitzsimons E. Correlates of mental illness and wellbeing in children: are they the same? Results from the UK millennium cohort study. J Am Acad Child Adolesc Psychiatry 2016;55:771–83. 10.1016/j.jaac.2016.05.019 27566118

[61] Lodebo BT, Möller J, Larsson J-O, et al. Socioeconomic position and self-harm among adolescents: a population-based cohort study in Stockholm, Sweden. Child Adolesc Psychiatry Ment Health 2017;11:46. 10.1186/s13034-017-0184-1 28878818 PMC5585967

[62] Reiss F. Socioeconomic inequalities and mental health problems in children and adolescents: a systematic review. Soc Sci Med 2013;90:24–31. 10.1016/j.socscimed.2013.04.026 23746605

[63] NISRA. Religion in Northern Ireland. 2011. Available: https://wwwninis2nisragovuk/public/census2011analysis/religion/religionCommentarypdf

[64] NISRA. Main statistics for Northern Ireland statistical bulletin: ethnic group. 2022. Available: https://wwwnisragovuk/system/files/statistics/census-2021-main-statistics-for-northern-ireland-phase-1-statistical-bulletin-ethnic-grouppdf

