# Supplementary material for: Adolescent mental well-being, religion and family activities: a cross-sectional study (Northern Ireland Schools and Wellbeing Study)
Source: BMJ Open. 2023 Jun 22;13(6):e071999. doi: 10.1136/bmjopen-2023-071999 (PMC10314551; doi:10.1136/bmjopen-2023-071999)
Supplement: Supplementary data [file bmjopen-2023-071999supp001.pdf]

Northern Ireland Study of Adolescent Wellbeing (NISAW)

Northern Ireland Study of Adolescent Wellbeing (NISAW)

CODING FILE

By completing this questionnaire, you are helping us to understand how and when adolescents seek help when they experience emotional difficulties. This questionnaire will be completed by many young people in Northern Ireland aged 13 to 16.

No-one outside the research team will see your answers. All the information you give will be kept completely confidential. By completing the questionnaire you confirm that you understand the participant information provided and give your consent to take part in this study.

Please read each question carefully before answering. We are interested in hearing *your* thoughts and feelings so there is no 'right' or 'wrong' way to answer these questions. The questionnaire should take approximately 25 minutes to complete.
Thank you for your contribution!

Initials (first *two* letters of *both* first *and* last name)**:**______________
Class: __________________________________

SCPID:____________________
Date:____________________

University of ULSTER
The Bamford Centre for Mental Health and Wellbeing
aware DEFEAT DEPRESSION

Northern Ireland Study of Adolescent Wellbeing (NISAW)

1.0 Some questions about you:
This first set of questions is simply to gather a bit of information about your background. Please tick the box that applies to you. If no box is provided simply write in your response. ITEMS 1.8-1.11 FAMILY AFFLUENCE SCALE. HIGH SCORE IS POSITIVE.

1.1	**Age**	☐ 13 ☐ 14 ☐ 15 ☐ 16	
1.2	**Gender**	☐ Male1<br>☐ Female2	
1.3	**How would you describe your Ethnic group?**	☐ White European1<br>☐ Bangladeshi3<br>☐ Black Africans5<br>☐ Black Caribbean7<br>☐ Black other9<br>☐ Chinese11<br>☐ Other_________________13	☐ Indian2<br>☐ Irish traveler community4<br>☐ Pakistani6<br>☐ Polish8<br>☐ Portuguese10<br>☐ Mixed12
1.4	**What is your Religion?**	☐ Catholic1<br>☐ Church of England2<br>☐ Presbyterian3<br>☐ Methodist4<br>☐ Baptist5<br>☐ Protestant (other) 6<br>☐ Other_________________13	☐ Hindu7<br>☐ Jewish8<br>☐ Buddhist9<br>☐ Muslim10<br>☐ Agnostic11<br>☐ Atheist/No beliefs12
1.5	**How important is religion to your family?**	☐ Very important1<br>☐ Moderately important2<br>☐ Not important3	
1.6	**How would you describe where you live?**	☐ I live in the city1<br>☐ I live in a town2<br>☐ I live in a village3<br>☐ I live in the country4	
1.7	**Do you currently live with your parents, including adoptive parents?**	☐ Yes, with my mother and my father in the same household1<br>☐ Yes, with my mother, but not my father2<br>☐ Yes, with my father, but not my mother3<br>☐ Yes, with my mother for some of the time, and with my father for some of the time4<br>☐ Other *(Please specify):* 5 ______________________________	
1.8	[redacted]	[redacted]	

University of ULSTER
The Bamford Centre for Mental Health and Wellbeing
aware DEFEAT DEPRESSION

Northern Ireland Study of Adolescent Wellbeing (NISAW)

1.9	[redacted]	[redacted]
1.10	[redacted]	[redacted]
1.11	[redacted]	[redacted]
1.12	**What is your father's occupation (job)?**	
1.13	**What is your mother's occupation (job)?**	

3.0 Your Family Life:
This section will help us understand factors of your family life such as your family activities and your relationship with your parents. Please tick **one** answer on **each line**. FAMILY LIFE (TAKEN FROM THE DASH STUDY). HIGH SCORES

**How often do you usually do each of these things with your family?**		**Every day**	**Most days**	**Weekly**	**Less than weekly**	**Never**
3.1	[redacted]	[redacted]	[redacted]	[redacted]	[redacted]	[redacted]
3.2	[redacted]	[redacted]	[redacted]	[redacted]	[redacted]	[redacted]
3.3	[redacted]	[redacted]	[redacted]	[redacted]	[redacted]	[redacted]
3.4	[redacted]	[redacted]	[redacted]	[redacted]	[redacted]	[redacted]
3.5	[redacted]	[redacted]	[redacted]	[redacted]	[redacted]	[redacted]
3.6	[redacted]	[redacted]	[redacted]	[redacted]	[redacted]	[redacted]

ARE POSITIVE.

Northern Ireland Study of Adolescent Wellbeing (NISAW)

5.0 Below are some statements about feelings and thoughts.

Please tick the box that best describes your experience of each **over the last 2 weeks.** WARWICK AND EDINBURGH MENTAL WELLBEING SCALE. HIGH SCORE IS POSITIVE.

	Statements	None of the time	Rarely	Some of the time	Often	All of the time
5.1	[redacted]	[redacted]	[redacted]	[redacted]	[redacted]	[redacted]
5.2	[redacted]	[redacted]	[redacted]	[redacted]	[redacted]	[redacted]
5.3	[redacted]	[redacted]	[redacted]	[redacted]	[redacted]	[redacted]
5.4	[redacted]	[redacted]	[redacted]	[redacted]	[redacted]	[redacted]
5.5	[redacted]	[redacted]	[redacted]	[redacted]	[redacted]	[redacted]
5.6	[redacted]	[redacted]	[redacted]	[redacted]	[redacted]	[redacted]
5.7	[redacted]	[redacted]	[redacted]	[redacted]	[redacted]	[redacted]
5.8	[redacted]	[redacted]	[redacted]	[redacted]	[redacted]	[redacted]
5.9	[redacted]	[redacted]	[redacted]	[redacted]	[redacted]	[redacted]
5.10	[redacted]	[redacted]	[redacted]	[redacted]	[redacted]	[redacted]
5.11	[redacted]	[redacted]	[redacted]	[redacted]	[redacted]	[redacted]
5.12	[redacted]	[redacted]	[redacted]	[redacted]	[redacted]	[redacted]
5.13	[redacted]	[redacted]	[redacted]	[redacted]	[redacted]	[redacted]
5.14	[redacted]	[redacted]	[redacted]	[redacted]	[redacted]	[redacted]

University of ULSTER

The Bamford Centre for Mental Health and Wellbeing

aware DEFEAT DEPRESSION

BMJ Publishing Group Limited (BMJ) disclaims all liability and responsibility arising from any reliance placed on this supplemental material which has been supplied by the author(s)

Northern Ireland Study of Adolescent Wellbeing (NISAW)

Thank you for spending the time to complete this questionnaire!

Before you hand in your questionnaire please check that you have not missed any questions!

We will make a random selection of 6-8 pupils to take part in a focus group to discuss the issues raised on this questionnaire or to talk about any issues related to mental health services for young people.

The focus groups are a great opportunity to make your voice heard.

If you require any further information contact details are provided on the information sheet. If any of the issues examined in this questionnaire have affected you, and you wish to receive further advice or support, help line contact details are also provided on the information sheet.

Northern Ireland Study of Adolescent Wellbeing (NISAW)

Questionnaire redacted material due to copyright:

1.8 -1.11 relates to the family affluence scale (1)

3.1-3.6 relates to the family activities (2)

5.1 – 5.14 relates to the Warwick-Edinburgh Mental Wellbeing Scale (3)

References:

1. Currie CE, Elton RA, Todd J, Platt S. Indicators of socioeconomic status for adolescents: the WHO Health Behaviour in School-aged Children Survey. Health education research. 1997;12(3):385-97.
2. Harding S, Read UM, Molaodi OR, Cassidy A, Maynard MJ, Lenguerrand E, et al. The Determinants of young Adult Social well-being and Health (DASH) study: diversity, psychosocial determinants and health. Social psychiatry and psychiatric epidemiology. 2015;50(8):1173-88.
3. Ng Fat L, Scholes S, Boniface S, Mindell J, Stewart-Brown S. Evaluating and establishing national norms for mental wellbeing using the short Warwick–Edinburgh Mental Well-being Scale (SWEMWBS): findings from the Health Survey for England. Quality of Life Research. 2017;26(5):1129-44.

AA:	T/M/B
ATT:	G/B
FSM:	Y/N
PC:	___________________________
SEN:	Y/N (____)
BI:	Y/N (____)
