# Supplementary material for: Adolescent mental well-being, religion and family activities: a cross-sectional study (Northern Ireland Schools and Wellbeing Study)
Source: BMJ Open. 2023 Jun 22;13(6):e071999. doi: 10.1136/bmjopen-2023-071999 (PMC10314551; doi:10.1136/bmjopen-2023-071999)
Supplement: Author's manuscript [file bmjopen-2023-071999.draft_revisions.pdf]

BMJ Open is committed to open peer review. As part of this commitment we make the peer review history of every article we publish publicly available.

When an article is published we post the peer reviewers' comments and the authors' responses online. We also post the versions of the paper that were used during peer review. These are the versions that the peer review comments apply to.

The versions of the paper that follow are the versions that were submitted during the peer review process. They are not the versions of record or the final published versions. They should not be cited or distributed as the published version of this manuscript.

BMJ Open is an open access journal and the full, final, typeset and author-corrected version of record of the manuscript is available on our site with no access controls, subscription charges or pay-per-view fees (http://bmjopen.bmj.com).

If you have any questions on BMJ Open's open peer review process please email info.bmjopen@bmj.com

BMJ Open

Adolescent mental wellbeing, religion and family bonding: a cross sectional study (Northern Ireland Schools and Wellbeing Study)

Journal:	*BMJ Open*
Manuscript ID	bmjopen-2023-071999
Article Type:	Original research
Date Submitted by the Author:	18-Jan-2023
Complete List of Authors:	Bamford, Jordan; The University of Manchester Division of Psychology and Mental Health,<br>Leavey, Gerard; University of Ulster, Bamford Centre for Mental Health and Wellbeing<br>Rosato, Michael; University of Ulster, Bamford Centre for Mental Health and Wellbeing<br>Divin, Natalie; Ulster University School of Psychology<br>Breslin, Gavin; Ulster University School of Psychology<br>Corry, Dagmar; Queen's University Belfast School of Psychology
Keywords:	MENTAL HEALTH, Child & adolescent psychiatry < PSYCHIATRY, PSYCHIATRY

SCHOLARONE™
Manuscripts

I, the Submitting Author has the right to grant and does grant on behalf of all authors of the Work (as defined in the below author licence), an exclusive licence and/or a non-exclusive licence for contributions from authors who are: i) UK Crown employees; ii) where BMJ has agreed a CC-BY licence shall apply, and/or iii) in accordance with the terms applicable for US Federal Government officers or employees acting as part of their official duties; on a worldwide, perpetual, irrevocable, royalty-free basis to BMJ Publishing Group Ltd ("BMJ") its licensees and where the relevant Journal is co-owned by BMJ to the co-owners of the Journal, to publish the Work in this journal and any other BMJ products and to exploit all rights, as set out in our licence.

The Submitting Author accepts and understands that any supply made under these terms is made by BMJ to the Submitting Author unless you are acting as an employee on behalf of your employer or a postgraduate student of an affiliated institution which is paying any applicable article publishing charge ("APC") for Open Access articles. Where the Submitting Author wishes to make the Work available on an Open Access basis (and intends to pay the relevant APC), the terms of reuse of such Open Access shall be governed by a Creative Commons licence – details of these licences and which Creative Commons licence will apply to this Work are set out in our licence referred to above.

Other than as permitted in any relevant BMJ Author's Self Archiving Policies, I confirm this Work has not been accepted for publication elsewhere, is not being considered for publication elsewhere and does not duplicate material already published. I confirm all authors consent to publication of this Work and authorise the granting of this licence.

For peer review only

Title:

Adolescent mental wellbeing, religion and family bonding: a cross sectional study (Northern Ireland Schools and Wellbeing Study)

Authors:

Jordan Bamford[1], Gerard Leavey[2]*, Michael Rosato[2], Natalie Divin[2], Gavin Breslin[2] and Dagmar Corry[3]

1 University of Manchester, Division of Psychology and Mental Health

2 The Bamford Centre for Mental Health & Wellbeing, Ulster University

3 Queen's University Belfast, School of Psychology

*Corresponding Author

Address: The Bamford Centre for Mental Health & Wellbeing, Ulster University, Cromore Road, Coleraine, Northern Ireland

Email: g.leavey@ulster.ac.uk

Abstract:

Objectives: Mental illness is increasing among adolescents, with greater demand for specialist services. In this study we sought to explore the relationship between adolescent mental wellbeing, religion and family bonding among a school based adolescent sample from Northern Ireland.

Setting: The Northern Ireland Schools and Wellbeing Study is a cross-sectional study (2014-2016) of pupils in Northern Ireland aged 13-18 years which included linked data from school administrative records.

Participants: 1618 adolescents from eight schools participated in this study.

Primary and secondary outcomes measures: Our primary outcome measure was The Warwick-Edinburgh Mental Well-being scale. We used linear regression and generated a series of incrementally adjusted models to explore the independent effects of a range of personal/social factors, including religious affiliation, importance of religion and family bonding.

Results: In our fully adjusted model, older adolescents and females reported lower mental wellbeing scores. Adolescents from more affluent backgrounds had greater mental wellbeing. We found no significant difference in mental wellbeing scores across religious groups. For adolescents where religion was very important compared to not important, they had greater mental wellbeing scores β 1.63 (95% CI 0.32, 2.95). Family bonding was associated with significant greater mental wellbeing scores, per unit increase in family bonding scale produced a 1.45% increase in mental wellbeing scores.

Conclusions: Our study indicates that non-religious adolescents may have lower mental wellbeing scores compared to their religious peers, irrespective of religious denomination. We relate this vulnerability to a possible sense of lack of firm identify and perceived marginalisation. Furthermore, adolescents with poor family bonding are more vulnerable to poor mental wellbeing. Policy should be mindful of how personal and social factors such as religion and family bonding can increase vulnerability to poor mental wellbeing for adolescents.

For peer review only - http://bmjopen.bmj.com/site/about/guidelines.xhtml

Strengths and limitations of this study:

- This study uses well-validated instruments to determine personal and social factors associated with mental wellbeing.
- This study uses a representative sample of adolescents from a range of schools
- A limitation is that the study is cross sectional, and therefore causality cannot be demonstrated.

Funding Statement:

This study was supported through Ulster University and the R&D Division of Northern Ireland Public Health Agency. The funders played no other role in the study and have not contributed to the analysis or writing up of the results which are the sole responsibility of the authors.

Funding reference number NA

Competing Interests:

All authors confirm that there are no conflicts of interest, perceived, real or potential.

Authorship:

Design and principal investigator (GL); data collection (DC, ND); data analysis (JB, GL and MR); writing (JB, GL, MR, DC, ND, GB)

All authors have agreed this manuscript and can confirm that there are no past, current or potential conflicts of interest.

For peer review only

Introduction

Adolescent mental health

Mental disorders among children and adolescents worldwide may be increasing; particularly cases of mood disorder, even before the COVID 19 pandemic (1-5). In England, an NHS survey found that 14.4% of 11-16 year olds had a mental disorder (5). There is evidence of increased demand for specialist mental health interventions for children and adolescents over the last decade (6). Moreover, there is evidence of increasing self-harming rates among certain groups of young people, including a 68% increase in self-harm rates among girls aged 13 to 16 since 2011 (7). In the UK, there is increasing numbers of reported suicide of those under the age of 18, mainly driven by an increased in girls aged 16 and boys aged 17 (8).

Adolescence is a critical life-course period for health, with mental health problems during adolescence having a much greater long-term cost than those developed during adulthood (9-11). The brain is particularly sensitive to stressors during this time (12). Social isolation, self-esteem and connectedness are important determinants of adolescent mental health (13). Associations between depressive symptoms and social media use have been identified, and this apparent vulnerability is greater for girls than boys (14). Further, among adolescents, gender, deprivation, child in need status, ethnicity and age all predict mental health outcomes (15, 16).

Mental Wellbeing and religion

Mental wellbeing is the positive aspect of mental health, and relates to a person's psychological functioning, life-satisfaction and ability to develop and maintain mutually benefiting relationships (17). The dual-continua model of mental health suggests that mental illness and mental wellbeing are not extreme ends of a single spectrum, rather they reflect distinct continua and influence one another via complex interrelationships (18). Mental wellbeing scores have been found to be positively associated with a broad range of mental and physical health outcomes (19). Therefore, understanding determinants of mental wellbeing is important.

Some evidence suggests that greater religiosity (mainly religious service attendance) is associated with greater wellbeing (20, 21), although most of this evidence is derived from predominantly Christian groups. One natural experimental study has suggested that greater involvement in religious activity (Ramadan fasting) increases wellbeing among Muslims (22). It is thought that religious attendance can bolster mental wellbeing by reducing loneliness, providing social support and engagement with community (23, 24) . Evidence from the United Kingdom has also indicated that more frequent religious service attendance improves wellbeing (25). However, religion and spirituality can be damaging to mental wellbeing too (26), in particular for vulnerable groups such as lesbian, gay or bisexual adolescents (27).

The context in Northern Ireland

Adolescent mental health in Northern Ireland has been a public concern following increased suicidality among young people (19, 28-30). Previous research suggests that post-conflict generations who have experienced violence in their communities may have higher levels of depression, psychotic symptoms and substance misuse (31). There is evidence that minority religious and atheist groups report poorer mental health and increased suicidality (29). Loneliness and poverty may also be associated with poor mental health in this population (32).

For peer review only - http://bmjopen.bmj.com/site/about/guidelines.xhtml

In this study we sought to explore the relationship between adolescent mental wellbeing, religion and family bonding. Such findings offer useful insight which can inform child and adolescent mental health services and guide policy.

Methodology

This was a cross-sectional study (2014-2016) of pupils in Northern Ireland aged 13-18 years which included linked data from school administrative records (29, 33). Eight schools were selected from a database of post-primary schools based on locale (urban/rural) and school type (e.g. Grammar or Secondary Modern). Grammar school attendance is based on selection through examination but generally acknowledged as having a strong middle-class bias (34). Within this study, parents were given a letter explaining the study, with an opt-out form for nonparticipation. All pupils provided written informed consent. Pupils completed the questionnaires in class groups following procedures used previously (35). Our sample comprised of 1618 young people who provided complete responses questions.

Measures

Outcome variable

Mental Well-being: The Warwick-Edinburgh Mental Well-being scale is used in NHS and private health services, and is generally one of the most widely used measures of mental wellbeing (36, 37). It contains 14 items covering psychological functioning and subjective wellbeing. Respondents respond to positively worded questions on a 5-point Likert scale. The responses are summed to provide a total score which varies between 14 to 70, with higher scores indicating higher levels of mental wellbeing. Overall, it is deemed a valid and reliable tool for measuring wellbeing in diverse populations (38, 39).

Religion measures

Religion group: Participants indicated religious group: this was summarised as catholic, protestant, other faith group, or atheist/agnostic.

Importance of religion: Participants were asked 'how important is religion to your family?' Responses were as follows: very important, moderately important or not important.

Family bonding

To assess family life we used the Family activities scale (40). A six item, five-point Likert scale self-report questionnaire (1=never to 5=every day). Items are summed, high scores indicate an integrated family life.

Confounders

We controlled for age (continuous), gender (male or female) and ethnicity (white European and other ethnic group). We controlled for school type (grammar and non-grammar) and family affluence. Family affluence was measured via the family affluence scale which examines socioeconomic inequalities. This measure is reliable and sensitive and validated for differentiating levels of affluence (41). It was examined as a continuous variable from 0 to 9, with greater scores indicating greater affluence. We also controlled for school rurality (rural and urban).

Analysis

For peer review only - http://bmjopen.bmj.com/site/about/guidelines.xhtml

Characteristics of the population were determined, and the relationship with characteristics of the population and mental wellbeing examined, with mean and standard deviation presented. To assess for significant differences between means, we used t-tests and ANOVAs if the variable was binary or multicategorical, respectively. For continuous variables, means and standard deviation presented, and we used linear regression and present the beta coefficient with associated p values. We used linear regression to explore the influence of religion, religious importance and family bonding on mental wellbeing. Fully adjusted odds ratios (ORs) and 95% confidence intervals are presented.

Results

Population characteristics are presented in Table 1. Our sample included adolescents aged 13 to 18, with over 90% of the group being 16 years or younger. This sample was mainly female (62.7%) and predominantly white European (94.7%). 57.5% of the sample attended non-grammar schools. In the family affluence scale, the average score was 6.0 (SD 1.7) and the average score for the family acts scale was 16.6 (SD 3.8). In this population, most adolescents identified as belonging to the catholic faith (60.4%) and 10.3% identified as being atheist/agnostic. Among this sample, over 70% of the adolescents included said that religion was either moderately or very important to their family. 60.3% of this sample of adolescents attended a school in an urban area.

The average score for mental wellbeing among the whole sample population was 47.7 (SD 9.7). Table 1 shows how the descriptive variables in this study relate to mental wellbeing. We identified no significant difference across age groups and ethnic groups with regards to mental wellbeing (unadjusted for other confounding predictors). We identified that across gender, religious groups, religious importance to family, rurality and school type, there were significant differences in mean scores for mental wellbeing (unadjusted for other confounding variables). We identified that females had on average lower mental wellbeing scores compared to males, 45.7 (SD 9.9) and 51.0 (SD 8.4) respectively. Lower wellbeing scores were found among atheist/agnostic adolescents, and those adolescents whose families did not consider religion to be important. Adolescents in grammar schools had lower on average mental wellbeing scores, as too did those adolescents attending rural schools. We identified that higher scores for both the family act scale and the family influence scale were associated with greater wellbeing scores for adolescents.

Table 2 shows the results of incrementally adjusted models for mental wellbeing. In the first model, before including variables related to religion and family bonding, we found that older adolescents and female adolescents had significantly lower scores for mental wellbeing. Those children with high scores with respect to family affluence had significantly higher scores for mental wellbeing. Interesting, neither grammar school status, rurality or ethnicity were significantly associated with predicting mental wellbeing scores. The inclusion of religious group, as seen in model 2, did not materially influence the previous significant associations of gender, age and affluence with mental wellbeing. We found that atheist adolescents compared to catholic children reported significantly lower scores for mental wellbeing β -2.98 (95% CI -4.57, -1.40). No significant differences between protestant and other religious adolescents compared to catholic adolescents was identified.

In model 3, we then included religious importance groupings. Like model 2, previous associations between mental wellbeing and age, gender and family affluence persisted. The association with non-religious adolescents and lower mental wellbeing was again evident, but the impact on wellbeing was reduced β -2.06 (95% CI -3.73, -0.40). Religious importance was associated with mental wellbeing. Adolescents whose family considered religion to be of moderate importance and very

For peer review only - http://bmjopen.bmj.com/site/about/guidelines.xhtml

important, compared to those adolescents where religion was of no importance, reported significantly greater mental wellbeing scores, with the highest scores seen in those adolescents where religion was very important β 2.56 (95% CI 1.19, 3.94).

Model 4 shows the final model which accounts for family bonding. Again, the influence of gender and age on mental wellbeing persisted, as too did family affluence (however beta coefficients were reduced). The inclusion of family bonding in this model influenced the association with religious groups and mental wellbeing. In our fully adjusted model, there were no significant difference in mental wellbeing scores across religious groups, including non-religious groups. For the religious importance category, only those adolescents where religion was very important compared to not important had greater mental wellbeing scores β 1.63 (95% CI 0.32, 2.95). There was no longer a significant difference between the not important and moderate important groups. Model 4 showed that the family bonding variable was associated with significant greater mental wellbeing scores, per unit increase in family bonding, this resulted in a 1.45% increase in mental wellbeing scores, β 0.78 (95% CI 0.67, 0.90)

Discussion

This study examined predictors of mental wellbeing among a school-based sample of adolescents, including religious and family bonding variables. We found that adolescents from families where religion was not important reported significantly lower mental wellbeing scores compared to their peers for whom religion was very important. This relationship has been identified in other studies which explored the influence of religious importance and its impact independent of religious affiliation (42). We initially identified that atheist/agnostic adolescents had lower mental wellbeing scores compared to other adolescent groups (catholic/protestant/other groups), however when we accounted for differences in family bonding, difference of mental wellbeing scores across religious groups were insignificant. Thus, where previous research has found non-religious groups having better mental health outcomes, this is not evident among our sample (43). Previous research using this sample has evidenced increased suicidality and mental illness for non-religious youth in Northern Ireland (29). This apparent vulnerability of non-religious adolescents may be related to challenges for this group. It may be considered that the social and psychological difficulties associated with being 'different' and the felt lack of firm identity compared to peers and sense of marginalization, negatively influences mental wellbeing. This experience of isolation and stigma for non-religious groups has been found to contribute to poorer mental health outcomes in the United States (44, 45).

Among our adolescent sample, females (compared to males) had lower wellbeing scores. Female vulnerability has been identified elsewhere, one study which examined adolescents in Mexico found that females after menarche had increased depressive symptoms, externalising problems and negative body image (46). Furthermore, another study identified that older female adolescents reported the lowest levels of self-compassion and lower emotional wellbeing scores (47). Differences in mental wellbeing between males and females have been related to societal gender inequality and cultural attitudes regarding gender (48).

We identified that family bonding was a key determinant of mental wellbeing, with greater bonding predicting greater mental wellbeing. Adolescents with greater bonding and more secure attachments have been found to have less engagement with high risk behaviours, fewer mental health problems, enhanced social skills and better coping strategies (49). Furthermore, some

For peer review only - http://bmjopen.bmj.com/site/about/guidelines.xhtml

evidence has suggested that parenting style and therefore bonding is a key determinant for emerging adult mental health, particularly for females (50).

We found that in our final model, family affluence remained a significant predictor for mental wellbeing scores. Interestingly, in our previous study, family affluence had little influence on mental health outcomes or trust in GPs for this sample of adolescents (29). Family affluence may therefore be an important determinant for mental wellbeing, but not mental health outcomes. Other research has shown that mental wellbeing and mental illness have distinct and different correlates, and that socioeconomic status and perceived socioeconomic status may correlate differently across the two (51). Other research has shown that socioeconomic position was associated with self-harm among adolescents, mainly for girls (52), and a systematic review found lower socioeconomic status a predictor for mental health problems (53).

Strengths and limitations

This study uses well-validated instruments to determine personal and social factors associated with mental wellbeing. This sample of adolescents from eight schools is a representative sample.

A limitation is that the study is cross sectional, and therefore causality cannot be demonstrated.

Conclusions:

This study indicates that religion and family bonding may be important determinants of adolescent mental wellbeing. We relate the identified vulnerability of those adolescents for whom religion is not important, to the experience of marginalisation and perceived lack of firm identity. Furthermore, our study indicates that adolescents with poor family bonding may be more vulnerable to poor mental wellbeing. Future policy should be mindful of personal and social factors which appear to be significant for adolescent mental wellbeing, and child and adolescent mental health services in Northern Ireland should consider the increased vulnerability of non-religious youth.

Ethics approval

The study was accorded a favourable opinion by the Ulster University Research Ethics Committee.

Ethics approval number UREC20/16 NISAW

For peer review only

For peer review only - http://bmjopen.bmj.com/site/about/guidelines.xhtml

Table 1: Sample characteristics and unadjusted distribution of mental wellbeing

		Total Population % (n)	WEMWBS Score Mean (SD)	Significance
Age	13	11.5 (186)	50.1 (8.5)	0.203
	14	30.4 (492)	48.4 (9.5)	
	15	25.7 (416)	47.1 (10.3)	
	16	24.4 (395)	46.9 (9.8)	
	17	7.2 (116)	46.0 (9.4)	
	18	0.8 (13)	47.6 (8.6)	
Gender	Male	37.3 (603)	51.0 (8.4)	<0.001
	Female	62.7 (1015)	45.7 (9.9)	
Ethnicity	White European	94.7 (1532)	47.7 (9.8)	0.188
	Other	5.3 (86)	47.9 (8.7)	
Religious group	Catholic	60.4 (978)	47.8 (9.6)	<0.001
	Protestant	27.1 (439)	48.4 (9.4)	
	Other	2.1 (34)	46.1 (11.3)	
	Atheist/Agnostic	10.3 (167)	45.4 (10.8)	
Religious Importance	Very Important	19.8 (321)	49.3 (10.1)	0.030
	Moderately Important	51.2 (829)	48.0 (9.1)	
	Not Important	28.9 (468)	46.1 (10.3)	
School type	Grammar	42.5 (688)	46.7 (10.1)	0.004
	Non-grammar	57.5 (930)	48.4 (9.4)	
Rurality	Rural	39.7 (643)	47.0 (10.0)	<0.001
	Urban	60.3 (975)	48.8 (9.3)	
Continuous Variables				
		Mean (SD)	Beta coefficient	P value
Family acts	Range 6 – 30	16.6 (3.8)	0.88	<0.001
Family affluence	Range 0 – 9	6.0 (1.7)	0.63	<0.001
Mental Wellbeing	Range 14 – 70	47.7 (9.7)	-	-

For peer review only - http://bmjopen.bmj.com/site/about/guidelines.xhtml

Table 2: Mental wellbeing in incrementally adjusted models

		M1 Beta coefficient (95% confidence intervals)	M2 Beta coefficient (95% confidence intervals)	M3 Beta coefficient (95% confidence intervals)	M4 Beta coefficient (95% confidence intervals)
Age		**-0.82 (-1.23, -0.42)****	**-0.75 (-1.16, -0.34)****	**-0.76 (-0.17, -0.35)****	**-0.45 (-0.84, -0.06)***
Gender	Male	1.00	1.00	1.00	1.00
	Female	**-5.21 (-6.17, -4.25)****	**-5.41 (-6.37, -4.44)****	**-5.38 (-6.34, -4.41)****	**-5.25 (-6.16, -4.33)****
Ethnicity	White European	1.00	1.00	1.00	1.00
	Other	-0.64 (-2.66, 1.39)	-0.23 (-2.27, 1.81)	-0.32 (-2.35, 1.72)	-0.29 (-2.22, 1.65)
School Type	Grammar	1.00	1.00	1.00	1.00
	Non-grammar	0.00 (-1.37, 1.38)	0.79 (-1.00, 2.58)	0.94 (-0.85, 2.73)	1.23 (-0.46, 2.93)
Family Affluence		**0.76 (0.48, 1.03)****	**0.75 (0.48, 1.02)****	**0.70 (0.43, 0.97)****	**0.41 (0.14, 0.67)***
Rurality	Urban	1.00	1.00	1.00	1.00
	Rural	0.99 (-0.31, 2.29)	0.31 (-1.19, 1.80)	0.13 (-1.37, 1.63)	-0.61 (-2.04, 0.82)
Religion	Catholic		1.00	1.00	1.00
	Protestant		-0.82 (-2.23, 0.59)	-0.60 (-2.01, 0.81)	-0.83 (-2.17, 0.51)
	Other		-2.95 (-6.18, 0.28)	-3.05 (-6.28, 0.16)	-2.44 (-5.49, 0.62)
	Atheist		**-2.98 (-4.57, -1.40)****	**-2.06 (-3.73, -0.40)***	-1.01 (-2.60, 0.58)
Religious Importance	Not important			1.00	1.00
	Moderate important			**1.49 (0.37, 2.61)***	0.91 (-0.16, 1.9)
	Very important			**2.56 (1.19, 3.94)****	**1.63 (0.32, 2.95)***
Family bonding					**0.78 (0.67, 0.90)****

*<0.05 **<0.001

For peer review only - http://bmjopen.bmj.com/site/about/guidelines.xhtml

For peer review only

References

1. Racine N, McArthur BA, Cooke JE, Eirich R, Zhu J, Madigan S. Global prevalence of depressive and anxiety symptoms in children and adolescents during COVID-19: a meta-analysis. JAMA pediatrics. 2021;175(11):1142-50.
2. Dalsgaard S, Thorsteinsson E, Trabjerg BB, Schullehner J, Plana-Ripoll O, Brikell I, et al. Incidence rates and cumulative incidences of the full spectrum of diagnosed mental disorders in childhood and adolescence. JAMA psychiatry. 2020;77(2):155-64.
3. Lu W. Adolescent depression: national trends, risk factors, and healthcare disparities. American journal of health behavior. 2019;43(1):181-94.
4. Polanczyk GV, Salum GA, Sugaya LS, Caye A, Rohde LA. Annual research review: A meta-analysis of the worldwide prevalence of mental disorders in children and adolescents. Journal of child psychology and psychiatry. 2015;56(3):345-65.
5. Sadler K, Vizard T, Ford T, Marchesell F, Pearce N, Mandalia D, et al. Mental health of children and young people in England, 2017. 2018.
6. Sarginson J, Webb RT, Stocks SJ, Esmail A, Garg S, Ashcroft DM. Temporal trends in antidepressant prescribing to children in UK primary care, 2000–2015. Journal of affective disorders. 2017;210:312-8.
7. Morgan C, Webb RT, Carr MJ, Kontopantelis E, Green J, Chew-Graham CA, et al. Incidence, clinical management, and mortality risk following self harm among children and adolescents: cohort study in primary care. bmj. 2017;359.
8. Manchester Uo. The National Confidential Inquiry into Suicide and Safety in Mental Health. Annual Report: UK patient and general population data, 2009-2019, and real time surveillance data. https://sites.manchester.ac.uk/ncish/reports/annual-report-2022/2022 [cited 2023 17th January]. Available from: https://sites.manchester.ac.uk/ncish/reports/annual-report-2022/.
9. Lee FS, Heimer H, Giedd JN, Lein ES, Šestan N, Weinberger DR, et al. Adolescent mental health—opportunity and obligation. Science. 2014;346(6209):547-9.
10. Moore GF, Cox R, Evans RE, Hallingberg B, Hawkins J, Littlecott HJ, et al. School, peer and family relationships and adolescent substance use, subjective wellbeing and mental health symptoms in Wales: A cross sectional study. Child indicators research. 2018;11(6):1951-65.
11. Patton GC, Sawyer SM, Santelli JS, Ross DA, Afifi R, Allen NB, et al. Our future: a Lancet commission on adolescent health and wellbeing. The Lancet. 2016;387(10036):2423-78.
12. Romeo RD. The impact of stress on the structure of the adolescent brain: Implications for adolescent mental health. Brain research. 2017;1654:185-91.
13. Preston AJ, Rew L. Connectedness, self-esteem, and prosocial behaviors protect adolescent mental health following social isolation: a systematic review. Issues in Mental Health Nursing. 2022;43(1):32-41.
14. Kelly Y, Zilanawala A, Booker C, Sacker A. Social media use and adolescent mental health: Findings from the UK Millennium Cohort Study. EClinicalMedicine. 2018;6:59-68.
15. Deighton J, Lereya ST, Casey P, Patalay P, Humphrey N, Wolpert M. Prevalence of mental health problems in schools: poverty and other risk factors among 28 000 adolescents in England. The British Journal of Psychiatry. 2019;215(3):565-7.
16. Bøe T, Skogen JC, Sivertsen B, Hysing M, Petrie KJ, Dearing E, et al. Economic volatility in childhood and subsequent adolescent mental health problems: a longitudinal population-based study of adolescents. BMJ open. 2017;7(9):e017030.
17. Stewart-Brown S, Janmohamed K. Warwick-Edinburgh mental well-being scale. User guide Version. 2008;1.
18. Iasiello M, Van Agteren J. Mental health and/or mental illness: A scoping review of the evidence and implications of the dual-continua model of mental health. Evidence Base: A journal of evidence reviews in key policy areas. 2020(1):1-45.

For peer review only - http://bmjopen.bmj.com/site/about/guidelines.xhtml

19. McKay MT, Andretta JR, Cole JC, Clarke M. Socio-demographic predictors of well-being in United Kingdom adolescents, and the impact of well-being on a range of health-related outcomes. Psychiatry Research. 2020;285:112728.
20. VanderWeele TJ. Religion and health in Europe: cultures, countries, context. European journal of epidemiology. 2017;32(10):857-61.
21. Chen Y, VanderWeele TJ. Associations of religious upbringing with subsequent health and well-being from adolescence to young adulthood: an outcome-wide analysis. American journal of epidemiology. 2018;187(11):2355-64.
22. Campante F, Yanagizawa-Drott D. Does religion affect economic growth and happiness? Evidence from Ramadan. The Quarterly Journal of Economics. 2015;130(2):615-58.
23. Lim C, Putnam RD. Religion, social networks, and life satisfaction. American sociological review. 2010;75(6):914-33.
24. Bhui K, King M, Dein S, O'Connor W. Ethnicity and religious coping with mental distress. Journal of mental health. 2008;17(2):141-51.
25. Aksoy O, Bann D, Fluharty ME, Nandi A. Religiosity and mental wellbeing among members of majority and minority religions: findings from Understanding Society: The UK Household Longitudinal Study. American journal of epidemiology. 2022;191(1):20-30.
26. Weber SR, Pargament KI. The role of religion and spirituality in mental health. Current opinion in psychiatry. 2014;27(5):358-63.
27. Page MJ, Lindahl KM, Malik NM. The role of religion and stress in sexual identity and mental health among lesbian, gay, and bisexual youth. Journal of Research on Adolescence. 2013;23(4):665-77.
28. McAlister S, Haydon D, Scraton P. Violence in the lives of children and youth in "post-conflict" Northern Ireland. Children Youth and Environments. 2013;23(1):1-22.
29. Leavey G, Rosato M, Harding S, Corry D, Divin N, Breslin G. Adolescent mental health problems, suicidality and seeking help from general practice: A cross-sectional study (Northern Ireland Schools and Wellbeing study). Journal of affective disorders. 2020;274:535-44.
30. Carlisle PA. "We don't talk about that around here": Religion, spirituality and mental health in Northern Ireland. Mental Health, Religion & Culture. 2015;18(5):396-407.
31. McAloney K, McCrystal P, Percy A, McCartan C. Damaged youth: prevalence of community violence exposure and implications for adolescent well-being in post-conflict Northern Ireland. Journal of Community Psychology. 2009;37(5):635-48.
32. Shevlin M, Murphy S, Mallett J, Stringer M, Murphy J. Adolescent loneliness and psychiatric morbidity in Northern Ireland. British journal of clinical psychology. 2013;52(2):230-4.
33. Corry DAS, Leavey G. Adolescent trust and primary care: Help-seeking for emotional and psychological difficulties. Journal of Adolescence. 2017;54:1-8.
34. Cribb J, Jesson D, Sibieta L, Skipp A, Vignoles A. Poor Grammar: Entry into Grammar Schools for Disadvantaged Pupils in England. Sutton Trust. 2013.
35. Leavey G, Rothi D, Paul R. Trust, autonomy and relationships: the help-seeking preferences of young people in secondary level schools in London (UK). Journal of adolescence. 2011;34(4):685-93.
36. Ng Fat L, Scholes S, Boniface S, Mindell J, Stewart-Brown S. Evaluating and establishing national norms for mental wellbeing using the short Warwick–Edinburgh Mental Well-being Scale (SWEMWBS): findings from the Health Survey for England. Quality of Life Research. 2017;26(5):1129-44.
37. Anthony R, Moore G, Page N, Hewitt G, Murphy S, Melendez-Torres G. Measurement invariance of the short Warwick-Edinburgh Mental Wellbeing Scale and latent mean differences (SWEMWBS) in young people by current care status. Quality of Life Research. 2022;31(1):205-13.
38. Stewart-Brown SL, Platt S, Tennant A, Maheswaran H, Parkinson J, Weich S, et al. The Warwick-Edinburgh Mental Well-being Scale (WEMWBS): a valid and reliable tool for measuring

For peer review only - http://bmjopen.bmj.com/site/about/guidelines.xhtml

mental well-being in diverse populations and projects. Journal of epidemiology and community health. 2011;65(Suppl 2):A38-A9.
39. Tennant R, Hiller L, Fishwick R, Platt S, Joseph S, Weich S, et al. The Warwick-Edinburgh mental well-being scale (WEMWBS): development and UK validation. Health and Quality of life Outcomes. 2007;5(1):1-13.
40. Harding S, Read UM, Molaodi OR, Cassidy A, Maynard MJ, Lenguerrand E, et al. The Determinants of young Adult Social well-being and Health (DASH) study: diversity, psychosocial determinants and health. Social psychiatry and psychiatric epidemiology. 2015;50(8):1173-88.
41. Currie CE, Elton RA, Todd J, Platt S. Indicators of socioeconomic status for adolescents: the WHO Health Behaviour in School-aged Children Survey. Health education research. 1997;12(3):385-97.
42. Green M, Elliott M. Religion, health, and psychological well-being. Journal of religion and health. 2010;49(2):149-63.
43. Baker JO, Stroope S, Walker MH. Secularity, religiosity, and health: Physical and mental health differences between atheists, agnostics, and nonaffiliated theists compared to religiously affiliated individuals. Social Science Research. 2018;75:44-57.
44. Brewster ME, Velez BL, Geiger EF, Sawyer JS. It's like herding cats: Atheist minority stress, group involvement, and psychological outcomes. Journal of Counseling Psychology. 2020;67(1):1.
45. Doane MJ, Elliott M. Perceptions of discrimination among atheists: Consequences for atheist identification, psychological and physical well-being. Psychology of Religion and Spirituality. 2015;7(2):130.
46. Benjet C, Hernandez-Guzman L. Gender differences in psychological well-being of Mexican early adolescents. Adolescence. 2001;36(141):47.
47. Bluth K, Campo RA, Futch WS, Gaylord SA. Age and gender differences in the associations of self-compassion and emotional well-being in a large adolescent sample. Journal of youth and adolescence. 2017;46(4):840-53.
48. Tesch-Römer C, Motel-Klingebiel A, Tomasik MJ. Gender differences in subjective well-being: Comparing societies with respect to gender equality. Social Indicators Research. 2008;85(2):329-49.
49. Moretti MM, Peled M. Adolescent-parent attachment: Bonds that support healthy development. Paediatrics & child health. 2004;9(8):551-5.
50. Steele EH, McKinney C. Emerging adult psychological problems and parenting style: Moderation by parent-child relationship quality. Personality and individual differences. 2019;146:201-8.
51. Patalay P, Fitzsimons E. Correlates of mental illness and wellbeing in children: are they the same? Results from the UK Millennium Cohort Study. Journal of the American Academy of Child & Adolescent Psychiatry. 2016;55(9):771-83.
52. Lodebo BT, Möller J, Larsson J-O, Engström K. Socioeconomic position and self-harm among adolescents: a population-based cohort study in Stockholm, Sweden. Child and adolescent psychiatry and mental health. 2017;11(1):1-9.
53. Reiss F. Socioeconomic inequalities and mental health problems in children and adolescents: a systematic review. Social science & medicine. 2013;90:24-31.

For peer review only - http://bmjopen.bmj.com/site/about/guidelines.xhtml

STROBE Statement—Checklist of items that should be included in reports of ***cross-sectional studies***

	Item No	Recommendation	Page No
**Title and abstract**	1	(*a*) Indicate the study's design with a commonly used term in the title or the abstract	1
		(*b*) Provide in the abstract an informative and balanced summary of what was done and what was found	1
**Introduction**			
Background/rationale	2	Explain the scientific background and rationale for the investigation being reported	3-4
Objectives	3	State specific objectives, including any prespecified hypotheses	4
**Methods**			
Study design	4	Present key elements of study design early in the paper	4
Setting	5	Describe the setting, locations, and relevant dates, including periods of recruitment, exposure, follow-up, and data collection	4
Participants	6	(*a*) Give the eligibility criteria, and the sources and methods of selection of participants	4
Variables	7	Clearly define all outcomes, exposures, predictors, potential confounders, and effect modifiers. Give diagnostic criteria, if applicable	4-5
Data sources/ measurement	8*	For each variable of interest, give sources of data and details of methods of assessment (measurement). Describe comparability of assessment methods if there is more than one group	*4-5*
Bias	9	Describe any efforts to address potential sources of bias	4-5
Study size	10	Explain how the study size was arrived at	4-5
Quantitative variables	11	Explain how quantitative variables were handled in the analyses. If applicable, describe which groupings were chosen and why	NA
Statistical methods	12	(*a*) Describe all statistical methods, including those used to control for confounding	4-5
		(*b*) Describe any methods used to examine subgroups and interactions	NA
		(*c*) Explain how missing data were addressed	NA
		(*d*) If applicable, describe analytical methods taking account of sampling strategy	4-5
		(*e*) Describe any sensitivity analyses	NA
**Results**			
Participants	13*	(a) Report numbers of individuals at each stage of study—eg numbers potentially eligible, examined for eligibility, confirmed eligible, included in the study, completing follow-up, and analysed	4-5
		(b) Give reasons for non-participation at each stage	NA
		(c) Consider use of a flow diagram	NA
Descriptive data	14*	(a) Give characteristics of study participants (eg demographic, clinical, social) and information on exposures and potential confounders	5, 8
		(b) Indicate number of participants with missing data for each variable of interest	NA
Outcome data	15*	Report numbers of outcome events or summary measures	5-6, 8-9

For peer review only

Main results	16	(*a*) Give unadjusted estimates and, if applicable, confounder-adjusted estimates and their precision (eg, 95% confidence interval). Make clear which confounders were adjusted for and why they were included	8-9
		(*b*) Report category boundaries when continuous variables were categorized	NA
		(*c*) If relevant, consider translating estimates of relative risk into absolute risk for a meaningful time period	NA
Other analyses	17	Report other analyses done—eg analyses of subgroups and interactions, and sensitivity analyses	NA
**Discussion**			
Key results	18	Summarise key results with reference to study objectives	6-7
Limitations	19	Discuss limitations of the study, taking into account sources of potential bias or imprecision. Discuss both direction and magnitude of any potential bias	6-7
Interpretation	20	Give a cautious overall interpretation of results considering objectives, limitations, multiplicity of analyses, results from similar studies, and other relevant evidence	6-7
Generalisability	21	Discuss the generalisability (external validity) of the study results	6-7
**Other information**			
Funding	22	Give the source of funding and the role of the funders for the present study and, if applicable, for the original study on which the present article is based	2

*Give information separately for exposed and unexposed groups.

Note: An Explanation and Elaboration article discusses each checklist item and gives methodological background and published examples of transparent reporting. The STROBE checklist is best used in conjunction with this article (freely available on the Web sites of PLoS Medicine at http://www.plosmedicine.org/, Annals of Internal Medicine at http://www.annals.org/, and Epidemiology at http://www.epidem.com/). Information on the STROBE Initiative is available at www.strobe-statement.org.

For peer review only

BMJ Open

Adolescent mental wellbeing, religion, and family activities: a cross-sectional study (Northern Ireland Schools and Wellbeing Study)

Journal:	*BMJ Open*
Manuscript ID	bmjopen-2023-071999.R1
Article Type:	Original research
Date Submitted by the Author:	20-Apr-2023
Complete List of Authors:	Bamford, Jordan; The University of Manchester Division of Psychology and Mental Health,<br>Leavey, Gerard; University of Ulster, Bamford Centre for Mental Health and Wellbeing<br>Rosato, Michael; University of Ulster, Bamford Centre for Mental Health and Wellbeing<br>Divin, Natalie; Ulster University School of Psychology<br>Breslin, Gavin; Ulster University School of Psychology<br>Corry, Dagmar; Queen's University Belfast School of Psychology
**Primary Subject Heading**:	Mental health
Secondary Subject Heading:	Mental health, Public health, Sociology
Keywords:	MENTAL HEALTH, Child & adolescent psychiatry < PSYCHIATRY, PSYCHIATRY

SCHOLARONE™
Manuscripts

I, the Submitting Author has the right to grant and does grant on behalf of all authors of the Work (as defined in the below author licence), an exclusive licence and/or a non-exclusive licence for contributions from authors who are: i) UK Crown employees; ii) where BMJ has agreed a CC-BY licence shall apply, and/or iii) in accordance with the terms applicable for US Federal Government officers or employees acting as part of their official duties; on a worldwide, perpetual, irrevocable, royalty-free basis to BMJ Publishing Group Ltd ("BMJ") its licensees and where the relevant Journal is co-owned by BMJ to the co-owners of the Journal, to publish the Work in this journal and any other BMJ products and to exploit all rights, as set out in our licence.

The Submitting Author accepts and understands that any supply made under these terms is made by BMJ to the Submitting Author unless you are acting as an employee on behalf of your employer or a postgraduate student of an affiliated institution which is paying any applicable article publishing charge ("APC") for Open Access articles. Where the Submitting Author wishes to make the Work available on an Open Access basis (and intends to pay the relevant APC), the terms of reuse of such Open Access shall be governed by a Creative Commons licence – details of these licences and which Creative Commons licence will apply to this Work are set out in our licence referred to above.

Other than as permitted in any relevant BMJ Author's Self Archiving Policies, I confirm this Work has not been accepted for publication elsewhere, is not being considered for publication elsewhere and does not duplicate material already published. I confirm all authors consent to publication of this Work and authorise the granting of this licence.

For peer review only

Adolescent mental wellbeing, religion, and family activities: a cross-sectional study (Northern Ireland Schools and Wellbeing Study)

Authors:

Jordan Bamford[1], Gerard Leavey[2]*, Michael Rosato[2], Natalie Divin[2], Gavin Breslin[2] and Dagmar Corry[3]

1. University of Manchester, Division of Psychology and Mental Health

2. The Bamford Centre for Mental Health & Wellbeing, Ulster University

3. Queen's University Belfast, School of Psychology

*Correspondence to:

Gerard Leavey
The Bamford Centre for Mental Health & Wellbeing, Ulster University, Cromore Road, Coleraine, Northern Ireland
Email: g.leavey@ulster.ac.uk

Abstract:

Objectives: In this study we seek to explore the relationship between adolescent mental wellbeing, religion and family activities among a school-based adolescent sample from Northern Ireland.

Setting: The Northern Ireland Schools and Wellbeing Study is a cross-sectional study (2014-2016) of pupils in Northern Ireland aged 13-18 years.

Participants: 1618 adolescents from eight schools participated in this study.

Outcomes measures: Our primary outcome measure was derived using the Warwick-Edinburgh Mental Wellbeing scale. We used hierarchical linear regression to explore the independent effects of a range of personal/social factors, including religious affiliation, importance of religion and family activities.

Results: In fully adjusted models, older adolescents and females reported lower mental wellbeing scores - for the year-on-year increase in age β=-0.45 (95%CI=-0.84, -0.06), and for females (compared to males) β=-5.25 (95%CI=-6.16, -4.33). More affluent adolescents reported better mental wellbeing. No significant differences in mental wellbeing scores across religious groups was found: compared to Catholics, Protestant adolescents recorded β=-0.83 (95%CI=-2.17, 0.51), other religious groups β=-2.44 (95%CI=-5.49, 0.62) and atheist adolescents β=-1.01 (95%CI=-2.60, 0.58). The importance of religion in the adolescents' lives was also tested: (compared to those for whom it was not important) those for whom it was very important had better mental wellbeing (β=1.63: 95%CI=0.32, 2.95). Higher levels of family activities were associated with higher mental wellbeing: each unit increase in family activity produced a 1.45% increase in the mental wellbeing score (β= 0.78: 95%CI=0.67, 0.90).

Conclusions: This study indicates that non-religious adolescents may have lower mental wellbeing scores when compared to their more religious peers, irrespective of religious denomination. This may relate to both a sense of lack of firm identity and perceived marginalisation. Additionally, adolescents with poor family cohesion are more vulnerable to poor mental wellbeing.

For peer review only - http://bmjopen.bmj.com/site/about/guidelines.xhtml

Strengths and limitations of this study

- This study uses well-validated instruments to determine personal and social factors associated with mental wellbeing.
- This study uses a representative sample of adolescents from a range of schools.
- A limitation is that the study is cross-sectional, and therefore causality cannot be demonstrated.

For peer review only

For peer review only - http://bmjopen.bmj.com/site/about/guidelines.xhtml

Introduction

Adolescent mental health

Worldwide, mental disorders - particularly mood disorders - among children and adolescents worldwide may be increasing, something noted even before the recent COVID-19 pandemic (1-5). In England, one National Health Service (NHS) survey found that 14.4% of 11-16 year olds reported a mental disorder (5), and evidence suggests increased demand over the last decade for specialist mental health interventions for children and adolescents (6). Additionally, there is evidence of increasing self-harm amongst young people, one study shows a 68% increase among girls aged 13-16 since 2011 (7). In the United Kingdom (UK), reported suicide has increased in those less than eighteen years of age, mainly driven by an increase in females aged sixteen years and males aged seventeen years (8).

Adolescence is a critical life-course period for health, with consequent mental health problems having a much greater long-term cost than those developed during adulthood (9-11). The brain is particularly sensitive to stressors during this time (12). Social isolation, self-esteem and connectedness are important determinants of adolescent mental health (13). Associations between depressive symptoms and social media use have been identified, and this apparent vulnerability is greater for girls than boys (14). Furthermore, among adolescents, sex, social deprivation, child-in-need status, ethnicity and age all predict poor mental health outcomes (15, 16).

Mental wellbeing, religion and family activities

Mental wellbeing is the positive aspect of mental health, relating to psychological functioning, life-satisfaction and ability to develop and maintain mutually beneficial relationships (17). The dual-continua model of mental health suggests that mental illness and mental wellbeing are not extreme ends of a single spectrum; but rather they reflect distinct continua which influence one another via complex interrelationships (18). Amongst adults, better mental wellbeing is associated with reduced risks of developing mental illness and improved chance of recovery from mental illness (19, 20). Evidence suggests that mental wellbeing is positively associated with a broad range of positive mental and physical health outcomes among adolescents in the UK (21).

Evidence suggests that greater religiosity (mainly religious service attendance) is associated with higher wellbeing among adults (22, 23), although this evidence is derived mainly from predominantly Christian groups. One *natural experiment* suggests that greater involvement in religious activity (Ramadan fasting) increases wellbeing among Muslims (24). Evidence suggests religiosity and spirituality are beneficial for adolescents: correlating with positive health attitudes and behaviours (25); and having a positive effect on psychological outcomes including risk behaviour, depression, wellbeing and self-esteem (26). It is thought that attendance at religious services can bolster mental wellbeing by reducing loneliness, providing social support and engagement with community (27, 28); and evidence from the UK also indicates that more frequent attendance improves wellbeing among adults (29). However, religion can be damaging to mental wellbeing (30), particularly for vulnerable groups such as lesbian, gay or bisexual adolescents (31). Another key determinant of mental wellbeing is within-family group activities, which evidence suggests is both an independent predictor of adolescent wellbeing (32), and a measure of family cohesion. The family has both a role in moderating risk factors for poor child development (33), and influences the building of resilience (34).

For peer review only - http://bmjopen.bmj.com/site/about/guidelines.xhtml

The context in Northern Ireland

Adolescent mental health in Northern Ireland (NI) is a public health concern following increased suicidality among young people (21, 35-37). Previous research suggests that post-conflict generations who have experienced violence in their communities may be at higher risk of depression, psychotic symptoms or substance misuse (38). Religious affiliation is of continuing social importance since the putative ending of the civil conflict (*The Troubles*). The most recent census data (from 2021) shows that NI comprises a small majority *Catholic* (45.7%) and large minority *Protestant* group (43.5%), with 1.5% identifying as *other non-Christian*, and the remaining 9.3% of the population identifying as *not belonging to any religion* - with this latter group increasing from 5.6% in 2011 (39). There is evidence that minority religious and atheist groups report poorer mental health and increased suicidality (36). Loneliness and poverty may also be associated with poor mental health in this population (40).

In this study we explore the relationship between adolescent mental wellbeing, religion, and family cohesion. Such findings offer useful insights to inform child and adolescent mental health services and guide policy.

Methods

Study design and setting

This is a cross-sectional study (2014-2016) of pupils in NI aged 13-18 years, based on a questionnaire and linked school administrative records data (36, 41). Eight schools were selected from a database of post-primary schools based on locale (urban/rural) and school type (*grammar* or *secondary modern*). While grammar school enrolment is based on selection (through examination) it is generally acknowledged to have a strong middle-class bias. In NI 43% of post-primary children attend grammar schools (42, 43). As part of the study protocol parents received a letter explaining the study, with an opt-out form for those requesting non-participation. All pupils provided written informed consent. Only five parents or children declined to participate. School absence was between 5% and 8% across the selected schools. Pupils completed the questionnaires in class groups following procedures described previously (44). A copy of the questionnaire is included as a Supplemental File. Our sample comprised of 1,618 young people who provided complete responses to the questionnaire.

Measures

Outcome variable

Mental Wellbeing: the Warwick-Edinburgh Mental Wellbeing scale is a widely used measures of mental wellbeing (45, 46), in both NHS and private health services. Overall, it is deemed a valid and reliable tool for measuring wellbeing in diverse populations (47, 48). It comprises fourteen items covering psychological functioning and subjective wellbeing, each a positively worded question on a 5-point Likert scale. The responses are summed to provide a total score which ranges between fourteen and seventy, with higher scores indicating better mental wellbeing.

Measures of religion

Religious group: participants indicated which religious group they identified with - summarised as *Catholic*, *Protestant*, *other faith group*, or *atheist/agnostic*.

Importance of religion: participants were asked about *how important is religion to your family* – with responses *very* important, *moderately* important or *not* important.

For peer review only - http://bmjopen.bmj.com/site/about/guidelines.xhtml

Family activities

To assess family cohesion we used the Family activities scale (49) - six questions, each a 5-point Likert scale (with responses 1=*never* to 5=*every day*). The responses are summed, with high scores indicating an integrated family life.

Confounders

We controlled for age (continuous) and ethnicity (white European, other ethnic group). Sex (male, female) – rather than gender - was recorded, as requested by school principals. We controlled for school type (*grammar*, *non-grammar*) and family affluence - the latter measured using the family affluence scale which quantifies socioeconomic inequity. This measure is reliable, sensitive and validated for differentiating levels of affluence (50). It was used in analysis as a continuous variable (range=0 to 9), with higher scores indicating greater affluence. We also controlled for the locale of the school (*rural*, *urban*).

Analysis

Characteristics of the population were determined, and the relationship with characteristics of the population and mental wellbeing examined, with mean and standard deviation presented. To assess for significant differences between means, we used t-tests and ANOVAs (for binary or categorical variables respectively). For continuous variables, means and standard deviation are presented. We used hierarchical linear regression to explore the influence of religion, religious importance, and family activities on wellbeing - and present results as beta-coefficient, associated 95% Confidence Intervals and p-values.

Patient and public involvement

None.

Results

Population characteristics are presented in Table 1. The sample included adolescents aged thirteen to eighteen years, with over 90% aged sixteen years or younger. This sample was mainly female (62.7%); predominantly white European (94.7%); with 57.5% attending non-grammar schools. With *family affluence* the mean score was 6.0 (SD 1.7); while for *family activities* the mean was 16.6 (SD 3.8). Most adolescents identified as Catholic (60.4%) and 10.3% identified as being atheist/agnostic; and over 70% said that religion was either moderately or very important to their family. In this sample 60.3% of adolescents attended school in an urban area.

The mean score for mental wellbeing over the whole sample was 47.7 (SD 9.7). We identified no significant difference across age-group and ethnic group with regards to mental wellbeing (unadjusted for other confounding predictors). We identified that across the sexes, religious groups, religious importance to family, rurality and school type, there were significant differences in mean scores for mental wellbeing (unadjusted for other confounding variables). We identified that females had on average lower mental wellbeing scores compared to males, 45.7 (SD 9.9) and 51.0 (SD 8.4) respectively. Lower wellbeing scores were found among atheist/agnostic adolescents, and those adolescents whose families did not consider religion to be important. Adolescents in grammar

For peer review only - http://bmjopen.bmj.com/site/about/guidelines.xhtml

For peer review only

schools had lower on average mental wellbeing scores, as too did those adolescents attending rural schools. We identified that higher scores for both the family act scale and the family influence scale were associated with greater wellbeing scores for adolescents.

Table 2 presents results for four incrementally adjusted models (M1 to M4) examining the association between wellbeing and the selected risk factors. In M1 both older and female adolescents record significantly poorer mental wellbeing. More affluent adolescents recorded better mental wellbeing. Neither grammar school status, rurality or ethnicity were significantly associated with predicting mental wellbeing.

The addition of *religious group* (M2) did not materially influence the already established associations of sex, age and affluence with mental wellbeing. However, atheist adolescents (compared with Catholics) reported significantly poorer mental wellbeing (β=-2.98: 95%CI=-4.57, -1.40). No significant differences between the reference Catholics and Protestant or adolescents from other religious groups was identified.

With the inclusion of *importance of religion* (M3) the established associations between wellbeing and age, sex and family affluence persisted, and while the poorer wellbeing associated with non-religious adolescents was again evident its impact on poorer wellbeing was reduced - from β=-2.98 (95%CI=-4.57, -1.40) to β=-2.06 (95%CI=-3.73, -0.40) in M2 and M3 respectively. Adolescents whose family considered religion to be either moderately or very important were (compared to those for whom religion was of no importance), reported significantly better wellbeing, for example β=2.56 (95%CI 1.19, 3.94), seen in those for whom religion was very important.

Finally, M4 - adding family activity levels – shows the fully adjusted model. Again, the influence of sex and age persists, as too does family affluence (though with reduced beta-coefficients). Inclusion of *family activity levels* also: (a) obviates any association between *all religious denomination* and wellbeing - including the non-religious group; and (b) constrains any beneficial effects of the *importance of religion*, and only those adolescents where religion was very important retain any beneficial effect (β=1.63: 95%CI=0.32, 2.95). Family activity levels is associated with significant higher mental wellbeing - β=0.78 (95%CI=0.67, 0.90), a per-unit increase of 1.45% wellbeing.

Discussion

In this study, examining predictors of mental wellbeing in a school-based sample of adolescents, we found that in families where religion was not important, there were poorer adolescent wellbeing outcomes scores compared to their peers for whom religion was very important. This relationship has been identified in other studies which explored the influence of religious importance and its impact independent of religious affiliation (51). We initially identified that atheist/agnostic adolescents had lower mental wellbeing scores compared to other adolescent groups (catholic/protestant/other groups), however when we accounted for differences in family activities, difference of mental wellbeing scores across religious groups were insignificant. Thus, where previous research has found non-religious groups having better mental health outcomes, this is not evident among our sample (52). Previous research using this sample found evidence of increased suicidality and mental illness associated with non-religious adolescents (36) - this apparent vulnerability may relate to social and psychological challenges associated with both being *different* and a felt lack of firm identity. This experience of marginalisation associated with non-religious groupings has been found to contribute to poorer mental health outcomes in the United States (53, 54).

For peer review only - http://bmjopen.bmj.com/site/about/guidelines.xhtml

In this sample females (compared to males) experienced poorer wellbeing: -female vulnerability has been identified elsewhere: one study of Mexican adolescents found that post-menarche females recorded increased depressive symptoms, externalising problems and negative body image (55); and another study identified that older female adolescents reported the lower levels of self-compassion and lower emotional wellbeing (56). Male/female differences in wellbeing have been related to societal cultural attitudes and inequity surrounding gender (57).

In this study more cohesive family activity levels were key to better mental wellbeing. Adolescents with secure attachments and cohesive families have been found to engage less in high risk behaviours, have fewer mental health problems, enhanced social skills and better coping strategies (58). Some evidence suggests that parenting style and, by implication, family activity levels plays a key role in emerging adult mental health, particularly for females (59).

In the fully adjusted model high levels of affluence was significantly associated with better wellbeing. In an earlier study using this data affluence had little influence on either mental health outcomes or trust in GPs (36). Family affluence may therefore be associated with mental wellbeing, but not necessarily mental health outcomes. Other research has shown that mental wellbeing and mental illness have distinct and different correlates, and that *actual* and *perceived* socioeconomic circumstance may correlate differently from each other (60). Other research has shown that socioeconomic position was associated with self-harm among adolescents, mainly for girls (61), and a systematic review found lower socioeconomic status associated with mental health problems (62).

Strengths and limitations

This study uses well-validated instruments to determine personal and social factors associated with mental wellbeing. This sample of adolescents from eight schools is relatively representative, with similar proportions of adolescents in grammar school education (43), and a similar proportion of atheist/agnostic adolescents as compared with the general NI population (39). While we note that Catholics are overrepresented in this sample (60% from a Catholic background, compared to 45.7% in the general population) (39) – current demographic trends in post-primary school enrolment record higher proportions of adolescent Catholics: in 2021/2022 50.2% of the grammar school population (32,990/65,313) and 51.7% (44,795/86,588) of the non-grammar school population were Catholic (43). Thie age-related redistribution of Catholics in relation to Protestants in NI mirrors current local demographic trends (63). Furthermore, our sample contains 5.3% of adolescents who do not belong to White European ethnicity. Census data shows that 3.4% of the population are from minority ethnic groups in Northern Ireland, and tend to be younger which is in keeping with our sample (64).

A limitation is that the study is cross-sectional, and therefore causality cannot be inferred. Further we recognise that the use of self-reported data has limitations, with social desirability bias possibly impacting on the results. Finally, as previously mentioned, we measured sex (not gender) at the request of school principals - we therefore acknowledge that this study cannot explore the influence of gender identity problems on mental wellbeing.

Conclusion

This study suggests that religious practice and family activities are important determinants of adolescent wellbeing. We highlight vulnerabilities associated with adolescents for whom religion is

For peer review only - http://bmjopen.bmj.com/site/about/guidelines.xhtml

not important, in relation to the experience of marginalisation and perceived lack of a firm identity. Additionally, the study suggests that less cohesive family units may also present vulnerabilities which can impact poor adolescent mental wellbeing. Future policy should be mindful of these personal and social factors, and child and adolescent mental health services in Northern Ireland should consider the increased vulnerability of non-religious youth.

*** ***

Ethics approval

The study was accorded a favourable opinion by the Ulster University Research Ethics Committee (Ethics approval number UREC20/16 NISAW).

Funding

This study was supported through Ulster University and the R&D Division of Northern Ireland Public Health Agency. The funders played no other role in the study and have not contributed to the analysis or writing up of the results which are the sole responsibility of the authors. Funding reference number: NA.

Competing interests

All authors confirm that there are no conflicts of interest, perceived, real or potential.

Contributors

Design and principal investigator (GL); data collection (DC, ND); data analysis (JB, GL and MR); writing (JB, GL, MR, DC, ND, GB). All authors have agreed this manuscript and can confirm that there are no past, current or potential conflicts of interest.

Data availability statement

No additional data available.

Table 1. Sample characteristics and unadjusted distribution of mental wellbeing

		Whole sample % (n)	WEMWBS score Mean (SD)	Significance
Age	13	11.5 (186)	50.1 (8.5)	0.203
	14	30.4 (492)	48.4 (9.5)	
	15	25.7 (416)	47.1 (10.3)	
	16	24.4 (395)	46.9 (9.8)	
	17	7.2 (116)	46.0 (9.4)	
	18	0.8 (13)	47.6 (8.6)	

For peer review only - http://bmjopen.bmj.com/site/about/guidelines.xhtml

Sex	Male<br>Female	37.3 (603)<br>62.7 (1015)	51.0 (8.4)<br>45.7 (9.9)	<0.001
Ethnicity	White European<br>Other	94.7 (1532)<br>5.3 (86)	47.7 (9.8)<br>47.9 (8.7)	0.188
Religious grouping	Catholic<br>Protestant<br>Other<br>Atheist/Agnostic	60.4 (978)<br>27.1 (439)<br>2.1 (34)<br>10.3 (167)	47.8 (9.6)<br>48.4 (9.4)<br>46.1 (11.3)<br>45.4 (10.8)	<0.001
Importance of religion	Very<br>Moderately<br>Unimportant	19.8 (321)<br>51.2 (829)<br>28.9 (468)	49.3 (10.1)<br>48.0 (9.1)<br>46.1 (10.3)	0.030
School type	Grammar<br>Non-grammar	42.5 (688)<br>57.5 (930)	46.7 (10.1)<br>48.4 (9.4)	0.004
Rurality	Rural<br>Urban	39.7 (643)<br>60.3 (975)	47.0 (10.0)<br>48.8 (9.3)	<0.001
Continuous variables				
		Mean (SD)	Beta coefficient	P-value
Family activities	Range 6 – 30	16.6 (3.8)	0.88	<0.001
Family affluence	Range 0 – 9	6.0 (1.7)	0.63	<0.001
Mental wellbeing	Range 14 – 70	47.7 (9.7)	-	-

For peer review only - http://bmjopen.bmj.com/site/about/guidelines.xhtml

Table 2. Relationship between mental wellbeing and included demographic and family characteristics

Data represents beta coefficients (and 95% CIs) from four incrementally developed regression models.

		M1<br>Beta coefficient (95% CI)	M2<br>Beta coefficient (95% CI)	M3<br>Beta coefficient (95% CI)	M4<br>Beta coefficient (95% CI)
Age$		**-0.82 (-1.23, -0.42)****	**-0.75 (-1.16, -0.34)****	**-0.76 (-0.17, -0.35)****	**-0.45 (-0.84, -0.06)***
Sex	Female (ref=Male)	**-5.21 (-6.17, -4.25)****	**-5.41 (-6.37, -4.44)****	**-5.38 (-6.34, -4.41)****	**-5.25 (-6.16, -4.33)****
Ethnicity	(ref=White European)<br>Other	-0.64 (-2.66, 1.39)	-0.23 (-2.27, 1.81)	-0.32 (-2.35, 1.72)	-0.29 (-2.22, 1.65)
School type	(ref=Grammar)<br>Non-grammar	0.00 (-1.37, 1.38)	0.79 (-1.00, 2.58)	0.94 (-0.85, 2.73)	1.23 (-0.46, 2.93)
Family affluence$		**0.76 (0.48, 1.03)****	**0.75 (0.48, 1.02)****	**0.70 (0.43, 0.97)****	**0.41 (0.14, 0.67)***
Locale of residence	Rural (ref=Urban)	0.99 (-0.31, 2.29)	0.31 (-1.19, 1.80)	0.13 (-1.37, 1.63)	-0.61 (-2.04, 0.82)
Religion	(ref=Catholic)<br>Protestant<br>Other<br>Atheist		-0.82 (-2.23, 0.59)<br>-2.95 (-6.18, 0.28)<br>**-2.98 (-4.57, -1.40)****	-0.60 (-2.01, 0.81)<br>-3.05 (-6.28, 0.16)<br>**-2.06 (-3.73, -0.40)***	-0.83 (-2.17, 0.51)<br>-2.44 (-5.49, 0.62)<br>-1.01 (-2.60, 0.58)
Importance of religion	(ref=Not important)<br>Moderately<br>Very			**1.49 (0.37, 2.61)***<br>**2.56 (1.19, 3.94)****	0.91 (-0.16, 1.9)<br>**1.63 (0.32, 2.95)***
Family activities$					**0.78 (0.67, 0.90)****

*=<0.05 **=<0.001.

For peer review only - http://bmjopen.bmj.com/site/about/guidelines.xhtml

$: variable treated as continuous.

For peer review only

For peer review only - http://bmjopen.bmj.com/site/about/guidelines.xhtml

References

1. Racine N, McArthur BA, Cooke JE, Eirich R, Zhu J, Madigan S. Global prevalence of depressive and anxiety symptoms in children and adolescents during COVID-19: a meta-analysis. JAMA pediatrics. 2021;175(11):1142-50.
2. Dalsgaard S, Thorsteinsson E, Trabjerg BB, Schullehner J, Plana-Ripoll O, Brikell I, et al. Incidence rates and cumulative incidences of the full spectrum of diagnosed mental disorders in childhood and adolescence. JAMA psychiatry. 2020;77(2):155-64.
3. Lu W. Adolescent depression: national trends, risk factors, and healthcare disparities. American journal of health behavior. 2019;43(1):181-94.
4. Polanczyk GV, Salum GA, Sugaya LS, Caye A, Rohde LA. Annual research review: A meta-analysis of the worldwide prevalence of mental disorders in children and adolescents. Journal of child psychology and psychiatry. 2015;56(3):345-65.
5. Sadler K, Vizard T, Ford T, Marchesell F, Pearce N, Mandalia D, et al. Mental health of children and young people in England, 2017. 2018.
6. Sarginson J, Webb RT, Stocks SJ, Esmail A, Garg S, Ashcroft DM. Temporal trends in antidepressant prescribing to children in UK primary care, 2000–2015. Journal of affective disorders. 2017;210:312-8.
7. Morgan C, Webb RT, Carr MJ, Kontopantelis E, Green J, Chew-Graham CA, et al. Incidence, clinical management, and mortality risk following self harm among children and adolescents: cohort study in primary care. bmj. 2017;359.
8. Manchester Uo. The National Confidential Inquiry into Suicide and Safety in Mental Health. Annual Report: UK patient and general population data, 2009-2019, and real time surveillance data. https://sites.manchester.ac.uk/ncish/reports/annual-report-2022/2022 [cited 2023 17th January]. Available from: https://sites.manchester.ac.uk/ncish/reports/annual-report-2022/.
9. Lee FS, Heimer H, Giedd JN, Lein ES, Šestan N, Weinberger DR, et al. Adolescent mental health—opportunity and obligation. Science. 2014;346(6209):547-9.
10. Moore GF, Cox R, Evans RE, Hallingberg B, Hawkins J, Littlecott HJ, et al. School, peer and family relationships and adolescent substance use, subjective wellbeing and mental health symptoms in Wales: A cross sectional study. Child indicators research. 2018;11(6):1951-65.
11. Patton GC, Sawyer SM, Santelli JS, Ross DA, Afifi R, Allen NB, et al. Our future: a Lancet commission on adolescent health and wellbeing. The Lancet. 2016;387(10036):2423-78.
12. Romeo RD. The impact of stress on the structure of the adolescent brain: Implications for adolescent mental health. Brain research. 2017;1654:185-91.
13. Preston AJ, Rew L. Connectedness, self-esteem, and prosocial behaviors protect adolescent mental health following social isolation: a systematic review. Issues in Mental Health Nursing. 2022;43(1):32-41.
14. Kelly Y, Zilanawala A, Booker C, Sacker A. Social media use and adolescent mental health: Findings from the UK Millennium Cohort Study. EClinicalMedicine. 2018;6:59-68.
15. Deighton J, Lereya ST, Casey P, Patalay P, Humphrey N, Wolpert M. Prevalence of mental health problems in schools: poverty and other risk factors among 28 000 adolescents in England. The British Journal of Psychiatry. 2019;215(3):565-7.
16. Bøe T, Skogen JC, Sivertsen B, Hysing M, Petrie KJ, Dearing E, et al. Economic volatility in childhood and subsequent adolescent mental health problems: a longitudinal population-based study of adolescents. BMJ open. 2017;7(9):e017030.
17. Stewart-Brown S, Janmohamed K. Warwick-Edinburgh mental well-being scale. User guide Version. 2008;1.
18. Iasiello M, Van Agteren J. Mental health and/or mental illness: A scoping review of the evidence and implications of the dual-continua model of mental health. Evidence Base: A journal of evidence reviews in key policy areas. 2020(1):1-45.
19. Keyes CL, Dhingra SS, Simoes EJ. Change in level of positive mental health as a predictor of future risk of mental illness. American journal of public health. 2010;100(12):2366-71.

For peer review only - http://bmjopen.bmj.com/site/about/guidelines.xhtml

20. Iasiello M, van Agteren J, Keyes CL, Cochrane EM. Positive mental health as a predictor of recovery from mental illness. Journal of Affective Disorders. 2019;251:227-30.
21. McKay MT, Andretta JR, Cole JC, Clarke M. Socio-demographic predictors of well-being in United Kingdom adolescents, and the impact of well-being on a range of health-related outcomes. Psychiatry Research. 2020;285:112728.
22. VanderWeele TJ. Religion and health in Europe: cultures, countries, context. European journal of epidemiology. 2017;32(10):857-61.
23. Chen Y, VanderWeele TJ. Associations of religious upbringing with subsequent health and well-being from adolescence to young adulthood: an outcome-wide analysis. American journal of epidemiology. 2018;187(11):2355-64.
24. Campante F, Yanagizawa-Drott D. Does religion affect economic growth and happiness? Evidence from Ramadan. The Quarterly Journal of Economics. 2015;130(2):615-58.
25. Rew L, Wong YJ. A systematic review of associations among religiosity/spirituality and adolescent health attitudes and behaviors. Journal of adolescent health. 2006;38(4):433-42.
26. Yonker JE, Schnabelrauch CA, DeHaan LG. The relationship between spirituality and religiosity on psychological outcomes in adolescents and emerging adults: A meta-analytic review. Journal of adolescence. 2012;35(2):299-314.
27. Lim C, Putnam RD. Religion, social networks, and life satisfaction. American sociological review. 2010;75(6):914-33.
28. Bhui K, King M, Dein S, O'Connor W. Ethnicity and religious coping with mental distress. Journal of mental health. 2008;17(2):141-51.
29. Aksoy O, Bann D, Fluharty ME, Nandi A. Religiosity and mental wellbeing among members of majority and minority religions: findings from Understanding Society: The UK Household Longitudinal Study. American journal of epidemiology. 2022;191(1):20-30.
30. Weber SR, Pargament KI. The role of religion and spirituality in mental health. Current opinion in psychiatry. 2014;27(5):358-63.
31. Page MJ, Lindahl KM, Malik NM. The role of religion and stress in sexual identity and mental health among lesbian, gay, and bisexual youth. Journal of Research on Adolescence. 2013;23(4):665-77.
32. Maynard MJ, Harding S. Ethnic differences in psychological well-being in adolescence in the context of time spent in family activities. Social psychiatry and psychiatric epidemiology. 2010;45:115-23.
33. Clarke-Stewart A, Dunn J. Families count: Effects on child and adolescent development: Cambridge University Press; 2006.
34. Luthar SS, Cicchetti D, Becker B. The construct of resilience: A critical evaluation and guidelines for future work. Child development. 2000;71(3):543-62.
35. McAlister S, Haydon D, Scraton P. Violence in the lives of children and youth in "post-conflict" Northern Ireland. Children Youth and Environments. 2013;23(1):1-22.
36. Leavey G, Rosato M, Harding S, Corry D, Divin N, Breslin G. Adolescent mental health problems, suicidality and seeking help from general practice: A cross-sectional study (Northern Ireland Schools and Wellbeing study). Journal of affective disorders. 2020;274:535-44.
37. Carlisle PA. "We don't talk about that around here": Religion, spirituality and mental health in Northern Ireland. Mental Health, Religion & Culture. 2015;18(5):396-407.
38. McAloney K, McCrystal P, Percy A, McCartan C. Damaged youth: prevalence of community violence exposure and implications for adolescent well-being in post-conflict Northern Ireland. Journal of Community Psychology. 2009;37(5):635-48.
39. NISRA. Main statistics for Northern Ireland Statistical bulletin: Religion. https://wwwnisragovuk/system/files/statistics/census-2021-main-statistics-for-northern-ireland-phase-1-statistical-bulletin-religionpdf. 2022.
40. Shevlin M, Murphy S, Mallett J, Stringer M, Murphy J. Adolescent loneliness and psychiatric morbidity in Northern Ireland. British journal of clinical psychology. 2013;52(2):230-4.

For peer review only - http://bmjopen.bmj.com/site/about/guidelines.xhtml

41. Corry DAS, Leavey G. Adolescent trust and primary care: Help-seeking for emotional and psychological difficulties. Journal of Adolescence. 2017;54:1-8.
42. Cribb J, Jesson D, Sibieta L, Skipp A, Vignoles A. Poor Grammar: Entry into Grammar Schools for Disadvantaged Pupils in England. Sutton Trust. 2013.
43. NISRA. Annual enrolments at schools and in funded pre-school education in Northern Ireland. https://wwweducation-nigovuk/sites/default/files/publications/education/Revised%2011th%20March%202022%20-%20Annual%20enrolments%20at%20schools%20and%20in%20funded%20pre-school%20education%20in%20Northern%20Ireland%2C%202021-22pdf. 2022.
44. Leavey G, Rothi D, Paul R. Trust, autonomy and relationships: the help-seeking preferences of young people in secondary level schools in London (UK). Journal of adolescence. 2011;34(4):685-93.
45. Ng Fat L, Scholes S, Boniface S, Mindell J, Stewart-Brown S. Evaluating and establishing national norms for mental wellbeing using the short Warwick–Edinburgh Mental Well-being Scale (SWEMWBS): findings from the Health Survey for England. Quality of Life Research. 2017;26(5):1129-44.
46. Anthony R, Moore G, Page N, Hewitt G, Murphy S, Melendez-Torres G. Measurement invariance of the short Warwick-Edinburgh Mental Wellbeing Scale and latent mean differences (SWEMWBS) in young people by current care status. Quality of Life Research. 2022;31(1):205-13.
47. Stewart-Brown SL, Platt S, Tennant A, Maheswaran H, Parkinson J, Weich S, et al. The Warwick-Edinburgh Mental Well-being Scale (WEMWBS): a valid and reliable tool for measuring mental well-being in diverse populations and projects. Journal of epidemiology and community health. 2011;65(Suppl 2):A38-A9.
48. Tennant R, Hiller L, Fishwick R, Platt S, Joseph S, Weich S, et al. The Warwick-Edinburgh mental well-being scale (WEMWBS): development and UK validation. Health and Quality of life Outcomes. 2007;5(1):1-13.
49. Harding S, Read UM, Molaodi OR, Cassidy A, Maynard MJ, Lenguerrand E, et al. The Determinants of young Adult Social well-being and Health (DASH) study: diversity, psychosocial determinants and health. Social psychiatry and psychiatric epidemiology. 2015;50(8):1173-88.
50. Currie CE, Elton RA, Todd J, Platt S. Indicators of socioeconomic status for adolescents: the WHO Health Behaviour in School-aged Children Survey. Health education research. 1997;12(3):385-97.
51. Green M, Elliott M. Religion, health, and psychological well-being. Journal of religion and health. 2010;49(2):149-63.
52. Baker JO, Stroope S, Walker MH. Secularity, religiosity, and health: Physical and mental health differences between atheists, agnostics, and nonaffiliated theists compared to religiously affiliated individuals. Social Science Research. 2018;75:44-57.
53. Brewster ME, Velez BL, Geiger EF, Sawyer JS. It's like herding cats: Atheist minority stress, group involvement, and psychological outcomes. Journal of Counseling Psychology. 2020;67(1):1.
54. Doane MJ, Elliott M. Perceptions of discrimination among atheists: Consequences for atheist identification, psychological and physical well-being. Psychology of Religion and Spirituality. 2015;7(2):130.
55. Benjet C, Hernandez-Guzman L. Gender differences in psychological well-being of Mexican early adolescents. Adolescence. 2001;36(141):47.
56. Bluth K, Campo RA, Futch WS, Gaylord SA. Age and gender differences in the associations of self-compassion and emotional well-being in a large adolescent sample. Journal of youth and adolescence. 2017;46(4):840-53.
57. Tesch-Römer C, Motel-Klingebiel A, Tomasik MJ. Gender differences in subjective well-being: Comparing societies with respect to gender equality. Social Indicators Research. 2008;85(2):329-49.
58. Moretti MM, Peled M. Adolescent-parent attachment: Bonds that support healthy development. Paediatrics & child health. 2004;9(8):551-5.

For peer review only - http://bmjopen.bmj.com/site/about/guidelines.xhtml

59. Steele EH, McKinney C. Emerging adult psychological problems and parenting style: Moderation by parent-child relationship quality. Personality and individual differences. 2019;146:201-8.
60. Patalay P, Fitzsimons E. Correlates of mental illness and wellbeing in children: are they the same? Results from the UK Millennium Cohort Study. Journal of the American Academy of Child & Adolescent Psychiatry. 2016;55(9):771-83.
61. Lodebo BT, Möller J, Larsson J-O, Engström K. Socioeconomic position and self-harm among adolescents: a population-based cohort study in Stockholm, Sweden. Child and adolescent psychiatry and mental health. 2017;11(1):1-9.
62. Reiss F. Socioeconomic inequalities and mental health problems in children and adolescents: a systematic review. Social science & medicine. 2013;90:24-31.
63. NISRA. Religion in Northern Ireland. https://wwwninis2nisragovuk/public/census2011analysis/religion/religionCommentarypdf. 2011.
64. NISRA. Main statistics for Northern Ireland Statistical bulletin: Ethnic group. https://wwwnisragovuk/system/files/statistics/census-2021-main-statistics-for-northern-ireland-phase-1-statistical-bulletin-ethnic-grouppdf. 2022.

For peer review only

Northern Ireland Study of Adolescent Wellbeing (NISAW)

CODING FILE

By completing this questionnaire, you are helping us to understand how and when adolescents seek help when they experience emotional difficulties. This questionnaire will be completed by many young people in Northern Ireland aged 13 to 16.

No-one outside the research team will see your answers. All the information you give will be kept completely confidential. By completing the questionnaire you confirm that you understand the participant information provided and give your consent to take part in this study.

Please read each question carefully before answering. We are interested in hearing *your* thoughts and feelings so there is no 'right' or 'wrong' way to answer these questions. The questionnaire should take approximately 25 minutes to complete.
Thank you for your contribution!

Initials (first *two* letters of *both* first *and* last name):____________________

Class: ____

SCPID:_________________________

Date:__________________________

University of ULSTER   The Bamford Centre for Mental Health and Wellbeing   aware DEFEAT DEPRESSION

For peer review only - http://bmjopen.bmj.com/site/about/guidelines.xhtml

Northern Ireland Study of Adolescent Wellbeing (NISAW)

1.0 Some questions about you:
This first set of questions is simply to gather a bit of information about your background. Please tick the box that applies to you. If no box is provided simply write in your response. ITEMS 1.8-1.11 FAMILY AFFLUENCE SCALE. HIGH SCORE IS POSITIVE.

1.1	**Age**	☐ 13 ☐ 14 ☐ 15 ☐ 16
1.2	**Gender**	☐ Male1<br>☐ Female2
1.3	**How would you describe your Ethnic group?**	☐ White European1 ☐ Indian2<br>☐ Bangladeshi3 ☐ Irish traveler community4<br>☐ Black African5 ☐ Pakistani6<br>☐ Black Caribbean7 ☐ Polish8<br>☐ Black other9 ☐ Portuguese10<br>☐ Chinese11 ☐ Mixed12<br>☐ Other________________13
1.4	**What is your Religion?**	☐ Catholic1 ☐ Hindu7<br>☐ Church of England2 ☐ Jewish8<br>☐ Presbyterian3 ☐ Buddhist9<br>☐ Methodist4 ☐ Muslim10<br>☐ Baptist5 ☐ Agnostic11<br>☐ Protestant (other) 6 ☐ Atheist/No beliefs12<br>☐ Other________________13
1.5	**How important is religion to your family?**	☐ Very important1<br>☐ Moderately important2<br>☐ Not important3
1.6	**How would you describe where you live?**	☐ I live in the city1<br>☐ I live in a town2<br>☐ I live in a village3<br>☐ I live in the country4
1.7	**Do you currently live with your parents, including adoptive parents?**	☐ Yes, with my mother and my father in the same household1<br>☐ Yes, with my mother, but not my father2<br>☐ Yes, with my father, but not my mother3<br>☐ Yes, with my mother for some of the time, and with my father for some of the time4<br>☐ Other *(Please specify)*: 5 ______________________________
1.8	[redacted]	[redacted]

University of ULSTER
The Bamford Centre for Mental Health and Wellbeing
aware DEFEAT DEPRESSION

For peer review only

Northern Ireland Study of Adolescent Wellbeing (NISAW)

1.9	[redacted]	[redacted]
1.10	[redacted]	[redacted]
1.11	[redacted]	[redacted]
1.12	**What is your father's occupation (job)?**	
1.13	**What is your mother's occupation (job)?**	

3.0 Your Family Life:
This section will help us understand factors of your family life such as your family activities and your relationship with your parents. Please tick **one** answer on **each line**. FAMILY LIFE (TAKEN FROM THE DASH STUDY). HIGH SCORES

**How often do you usually do each of these things with your family?**		**Every day**	**Most days**	**Weekly**	**Less than weekly**	**Never**
3.1	[redacted]	[redacted]	[redacted]	[redacted]	[redacted]	[redacted]
3.2	[redacted]	[redacted]	[redacted]	[redacted]	[redacted]	[redacted]
3.3	[redacted]	[redacted]	[redacted]	[redacted]	[redacted]	[redacted]
3.4	[redacted]	[redacted]	[redacted]	[redacted]	[redacted]	[redacted]
3.5	[redacted]	[redacted]	[redacted]	[redacted]	[redacted]	[redacted]
3.6	[redacted]	[redacted]	[redacted]	[redacted]	[redacted]	[redacted]

ARE POSITIVE.

University of ULSTER

The Bamford Centre for Mental Health and Wellbeing

aware DEFEAT DEPRESSION

For peer review only

Northern Ireland Study of Adolescent Wellbeing (NISAW)

5.0 Below are some statements about feelings and thoughts.

Please tick the box that best describes your experience of each **over the last 2 weeks.** WARWICK AND EDINBURGH MENTAL WELLBEING SCALE. HIGH SCORE IS POSITIVE.

	Statements	None of the time	Rarely	Some of the time	Often	All of the time
5.1	[redacted]					
5.2	[redacted]					
5.3	[redacted]					
5.4	[redacted]					
5.5	[redacted]					
5.6	[redacted]					
5.7	[redacted]					
5.8	[redacted]					
5.9	[redacted]					
5.10	[redacted]					
5.11	[redacted]					
5.12	[redacted]					
5.13	[redacted]					
5.14	[redacted]					

For peer review only

For peer review only - http://bmjopen.bmj.com/site/about/guidelines.xhtml

Northern Ireland Study of Adolescent Wellbeing (NISAW)

Thank you for spending the time to complete this questionnaire!

Before you hand in your questionnaire please check that you have not missed any questions!

We will make a random selection of 6-8 pupils to take part in a focus group to discuss the issues raised on this questionnaire or to talk about any issues related to mental health services for young people.

The focus groups are a great opportunity to make your voice heard.

If you require any further information contact details are provided on the information sheet. If any of the issues examined in this questionnaire have affected you, and you wish to receive further advice or support, help line contact details are also provided on the information sheet.

University of ULSTER

The Bamford Centre for Mental Health and Wellbeing

aware DEFEAT DEPRESSION

Northern Ireland Study of Adolescent Wellbeing (NISAW)

Questionnaire redacted material due to copyright:

1.8 -1.11 relates to the family affluence scale (1)

3.1-3.6 relates to the family activities (2)

5.1 – 5.14 relates to the Warwick-Edinburgh Mental Wellbeing Scale (3)

References:
1. Currie CE, Elton RA, Todd J, Platt S. Indicators of socioeconomic status for adolescents: the WHO Health Behaviour in School-aged Children Survey. Health education research. 1997;12(3):385-97.
2. Harding S, Read UM, Molaodi OR, Cassidy A, Maynard MJ, Lenguerrand E, et al. The Determinants of young Adult Social well-being and Health (DASH) study: diversity, psychosocial determinants and health. Social psychiatry and psychiatric epidemiology. 2015;50(8):1173-88.
3. Ng Fat L, Scholes S, Boniface S, Mindell J, Stewart-Brown S. Evaluating and establishing national norms for mental wellbeing using the short Warwick–Edinburgh Mental Well-being Scale (SWEMWBS): findings from the Health Survey for England. Quality of Life Research. 2017;26(5):1129-44.

AA:	T/M/B
ATT:	G/B
FSM:	Y/N
PC:	____________________________
SEN:	Y/N (____)
BI:	Y/N (____)

For peer review only

For peer review only - http://bmjopen.bmj.com/site/about/guidelines.xhtml

STROBE Statement—Checklist of items that should be included in reports of ***cross-sectional studies***

	Item No	Recommendation	Page No
**Title and abstract**	1	(*a*) Indicate the study's design with a commonly used term in the title or the abstract	1
		(*b*) Provide in the abstract an informative and balanced summary of what was done and what was found	1
**Introduction**			
Background/rationale	2	Explain the scientific background and rationale for the investigation being reported	3-4
Objectives	3	State specific objectives, including any prespecified hypotheses	4
**Methods**			
Study design	4	Present key elements of study design early in the paper	4
Setting	5	Describe the setting, locations, and relevant dates, including periods of recruitment, exposure, follow-up, and data collection	4
Participants	6	(*a*) Give the eligibility criteria, and the sources and methods of selection of participants	4
Variables	7	Clearly define all outcomes, exposures, predictors, potential confounders, and effect modifiers. Give diagnostic criteria, if applicable	4-5
Data sources/ measurement	8*	For each variable of interest, give sources of data and details of methods of assessment (measurement). Describe comparability of assessment methods if there is more than one group	*4-5*
Bias	9	Describe any efforts to address potential sources of bias	4-5
Study size	10	Explain how the study size was arrived at	4-5
Quantitative variables	11	Explain how quantitative variables were handled in the analyses. If applicable, describe which groupings were chosen and why	NA
Statistical methods	12	(*a*) Describe all statistical methods, including those used to control for confounding	4-5
		(*b*) Describe any methods used to examine subgroups and interactions	NA
		(*c*) Explain how missing data were addressed	NA
		(*d*) If applicable, describe analytical methods taking account of sampling strategy	4-5
		(*e*) Describe any sensitivity analyses	NA
**Results**			
Participants	13*	(a) Report numbers of individuals at each stage of study—eg numbers potentially eligible, examined for eligibility, confirmed eligible, included in the study, completing follow-up, and analysed	4-5
		(b) Give reasons for non-participation at each stage	NA
		(c) Consider use of a flow diagram	NA
Descriptive data	14*	(a) Give characteristics of study participants (eg demographic, clinical, social) and information on exposures and potential confounders	5, 8
		(b) Indicate number of participants with missing data for each variable of interest	NA
Outcome data	15*	Report numbers of outcome events or summary measures	5-6, 8-9

For peer review only - http://bmjopen.bmj.com/site/about/guidelines.xhtml

Main results	16	(*a*) Give unadjusted estimates and, if applicable, confounder-adjusted estimates and their precision (eg, 95% confidence interval). Make clear which confounders were adjusted for and why they were included	8-9
		(*b*) Report category boundaries when continuous variables were categorized	NA
		(*c*) If relevant, consider translating estimates of relative risk into absolute risk for a meaningful time period	NA
Other analyses	17	Report other analyses done—eg analyses of subgroups and interactions, and sensitivity analyses	NA
**Discussion**			
Key results	18	Summarise key results with reference to study objectives	6-7
Limitations	19	Discuss limitations of the study, taking into account sources of potential bias or imprecision. Discuss both direction and magnitude of any potential bias	6-7
Interpretation	20	Give a cautious overall interpretation of results considering objectives, limitations, multiplicity of analyses, results from similar studies, and other relevant evidence	6-7
Generalisability	21	Discuss the generalisability (external validity) of the study results	6-7
**Other information**			
Funding	22	Give the source of funding and the role of the funders for the present study and, if applicable, for the original study on which the present article is based	2

*Give information separately for exposed and unexposed groups.

Note: An Explanation and Elaboration article discusses each checklist item and gives methodological background and published examples of transparent reporting. The STROBE checklist is best used in conjunction with this article (freely available on the Web sites of PLoS Medicine at http://www.plosmedicine.org/, Annals of Internal Medicine at http://www.annals.org/, and Epidemiology at http://www.epidem.com/). Information on the STROBE Initiative is available at www.strobe-statement.org.

For peer review only

For peer review only - http://bmjopen.bmj.com/site/about/guidelines.xhtml
